# Supplementary figures and images for: Engineering of Cas12a nuclease variants with enhanced genome-editing specificity
Source: PLoS Biol. 2024 Mar 14;22(3):e3002514. doi: 10.1371/journal.pbio.3002514 (PMC10965058; doi:10.1371/journal.pbio.3002514)

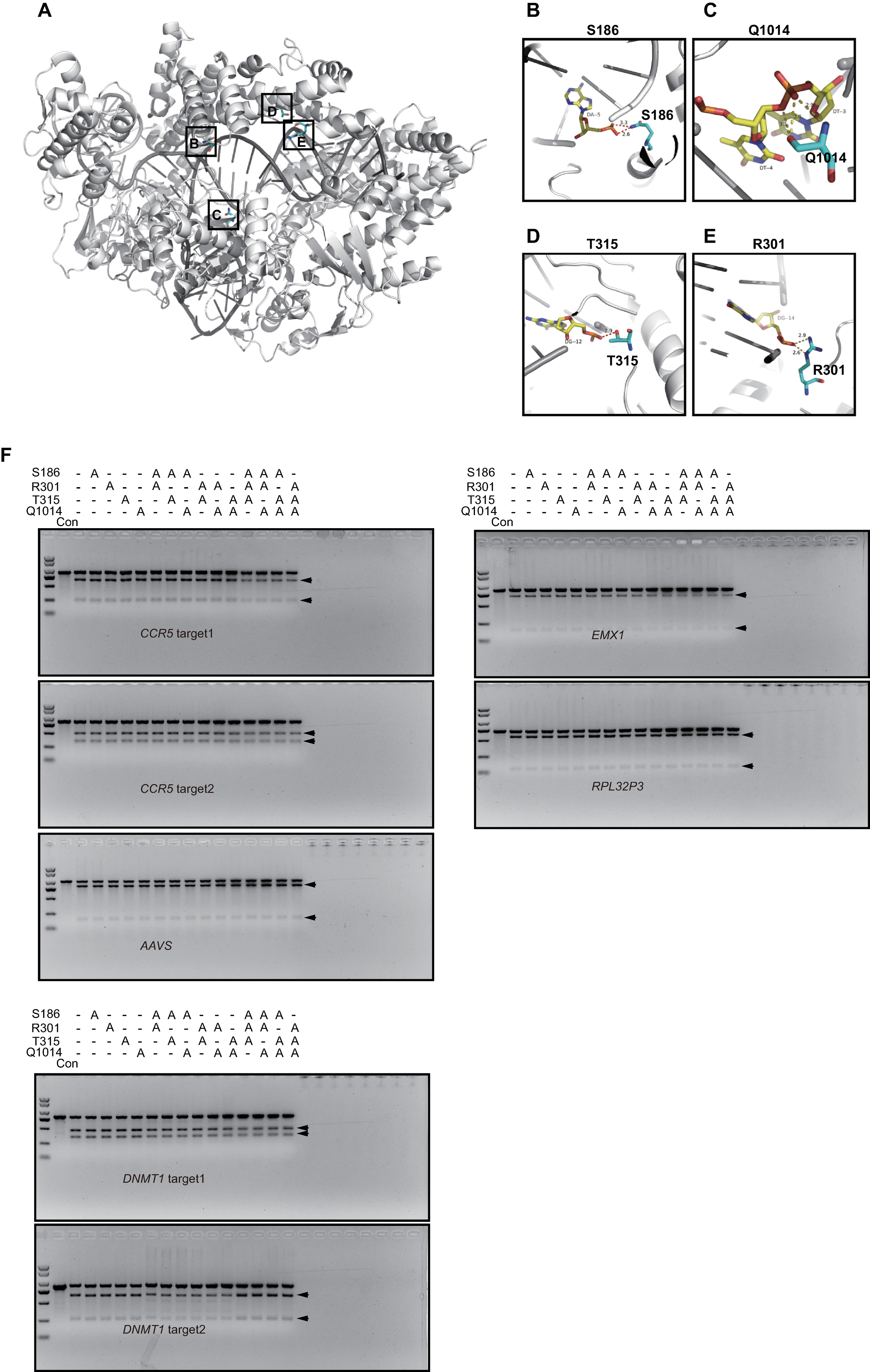

Supplement: S1 Fig — (A–E) Structural representations of AsCas12a-crRNA-DNA complex. In structural representations, amino acid residues (S186, R301, T315, and Q1014) that made direct hydrogen bonds to the phosphate backbone of the target DNA strand within a 3.0-Å distance. Boxes indicate regions shown in detail in B–E. Images generated from PDBID:5B43 (ref. [38]) visualized in PyMOL (v 1.8.6.0). (F) Activities of AsCas12a engineered variants bearing amino acid substitutions when tested against 7 endogenous sites in human cells. Activities assessed by T7E1 assay. (TIF) [file pbio.3002514.s001.tif]

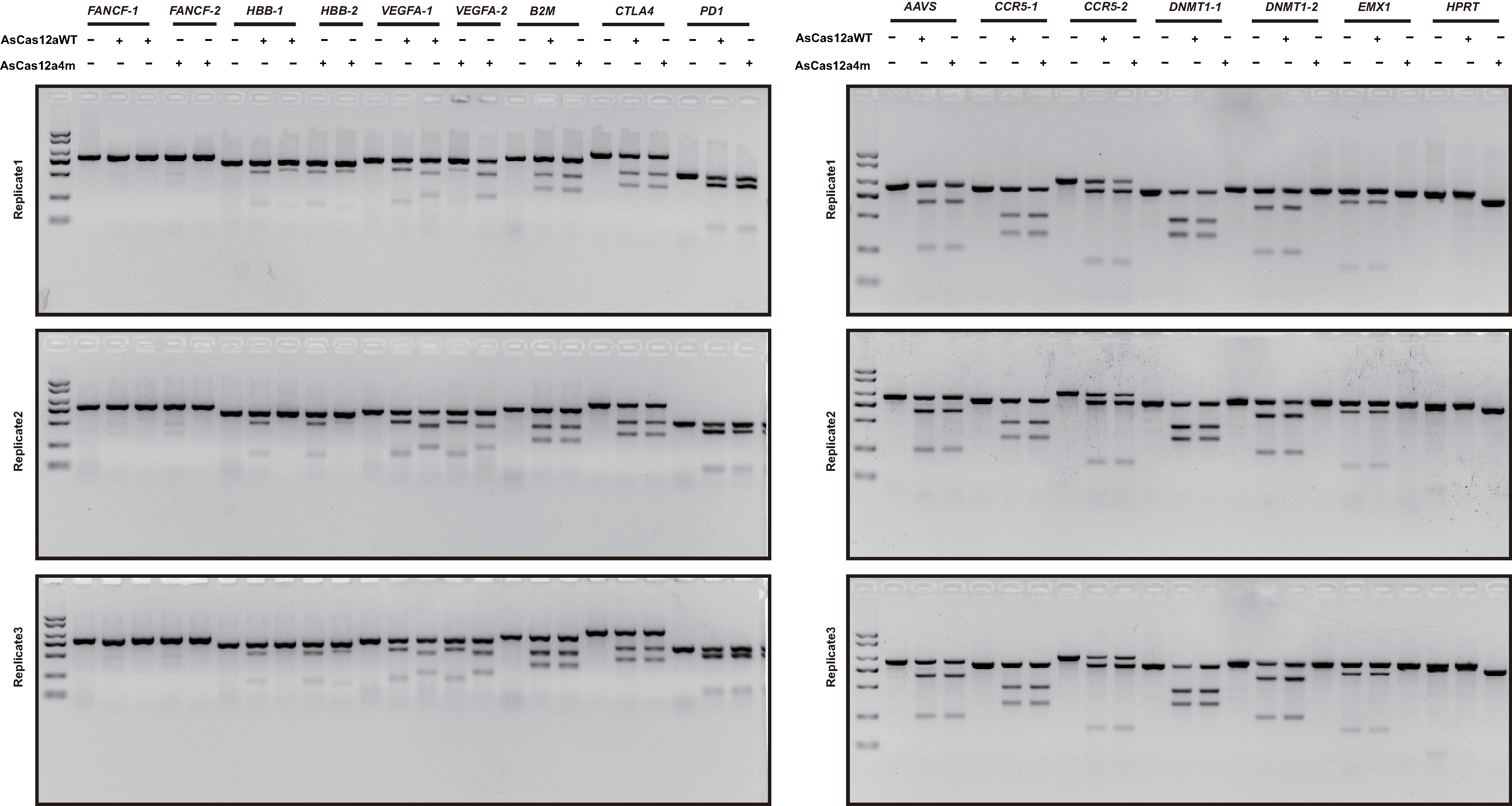

Supplement: S2 Fig — Comparative analysis of wild-type AsCas12a and AsCas12a variant (AsCas12a4m) with 16 sgRNAs targeting 11 genes. Full gel images of Fig 1C. Three independent transfection replicates were done, and activities assessed by T7E1 assay. (TIF) [file pbio.3002514.s002.tif]

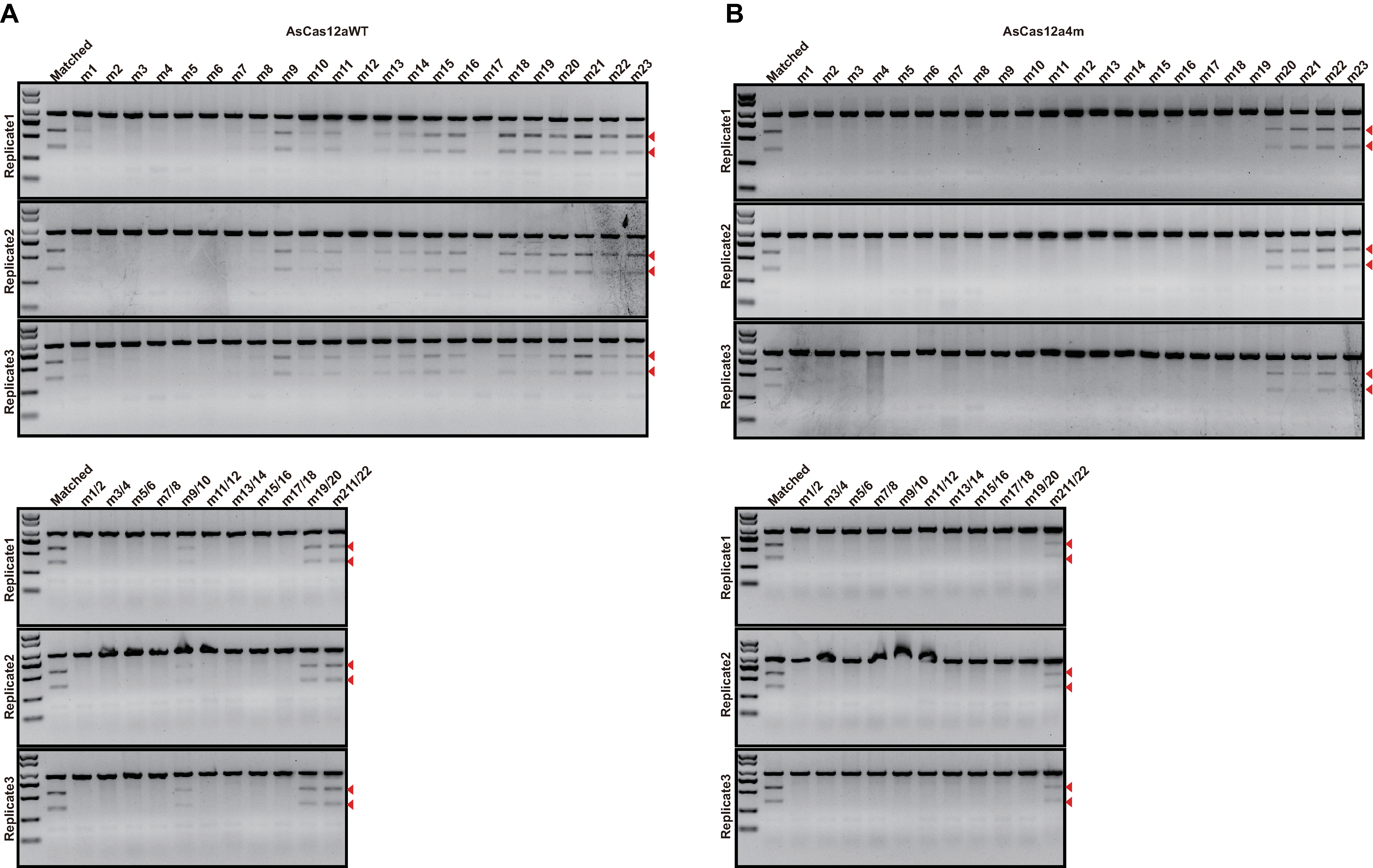

Supplement: S4 Fig — (A) Full gel image for AsCas12aWT activities with single mismatched crRNAs (top) and double mismatched crRNAs (bottom) toward B2M. (B) Full gel image for AsCas12a4m activities with single mismatched crRNAs (top) and double mismatched crRNAs (bottom) toward B2M. Three independent transfection replicates were done, and activities assessed by T7E1 assay. Arrows indicates cleavage products. (TIF) [file pbio.3002514.s004.tif]

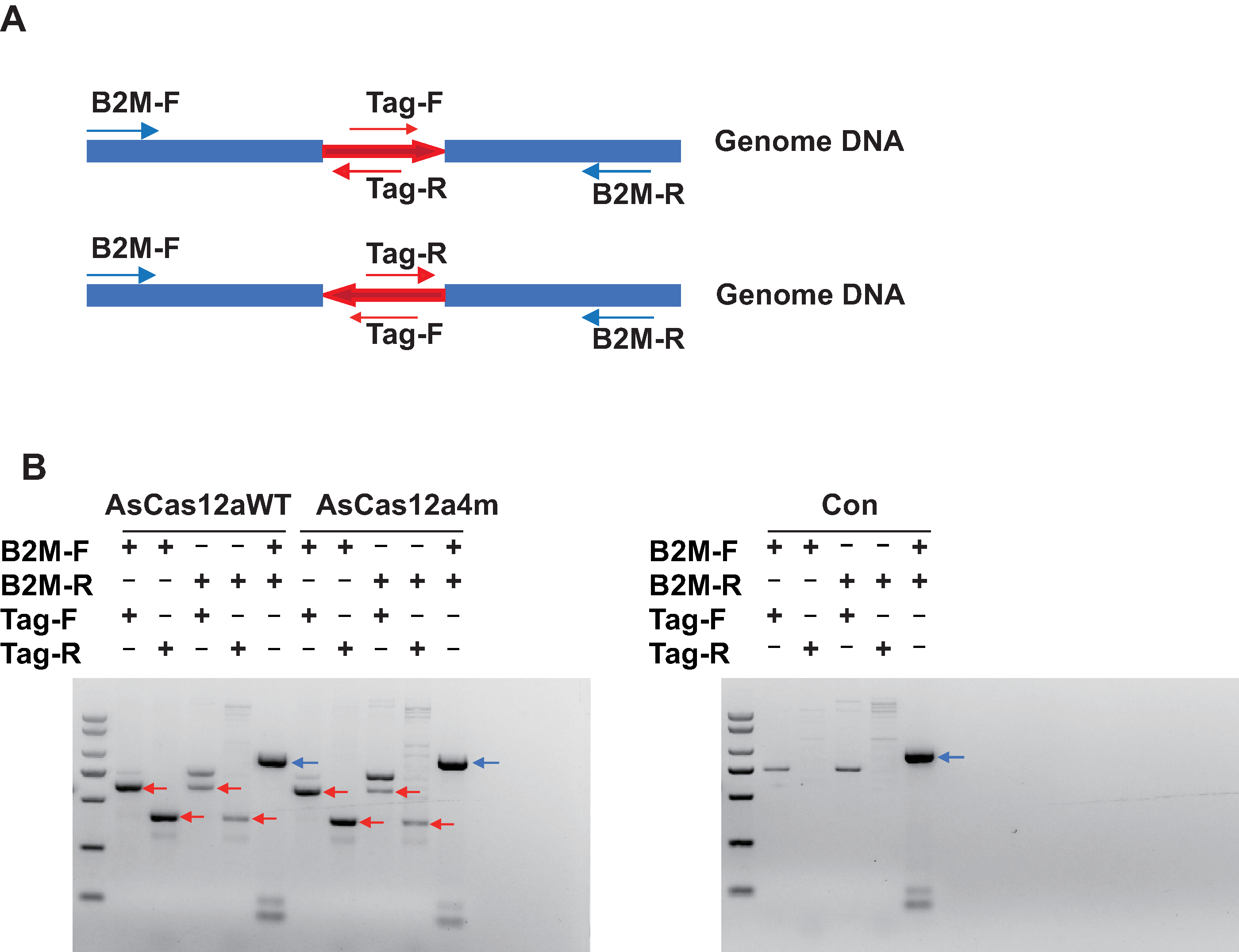

Supplement: S5 Fig — (A) Schematic of the Tag-PCR and Genome-PCR. Red arrows indicate genome-specific primers; blue arrows indicate tag-specific primers (Tag-F/R). (B) Full gel images of Tag-PCR and Genome-PCR. Red arrow bands indicate the PCR products using the genome-specific primers (B2M-F/R) with tag-specific primers (Tag-F/R). Blue arrow bands indicate the PCR products using the genome-specific primers B2M-F and B2M-R. Con means transfecting without As variants plasmids. (TIF) [file pbio.3002514.s005.tif]

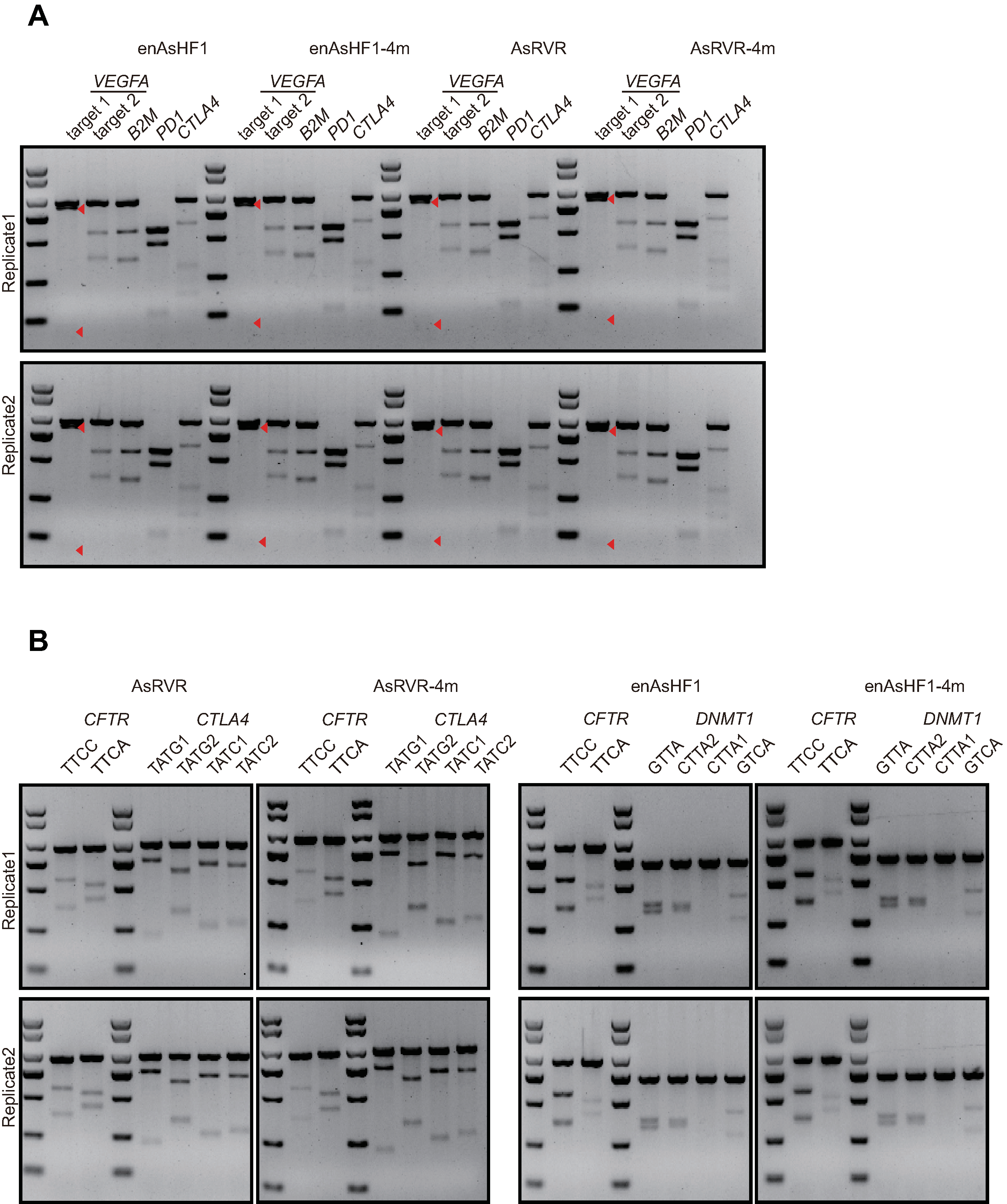

Supplement: S8 Fig — (A) Activity analysis of enAsHF1, AsRVR and enAsHF1-4m, AsRVR-4m with 5 crRNAs targeting 4 genes (VEGFA, B2M, PD1, CTLA4) at TTTV PAMs. Full gel images of Fig 3A and 3B, activity analysis of enAsHF1, AsRVR and enAsHF1-4m, AsRVR-4m with 10 crRNAs targeting 3 genes (CFTR, CTLA4, DNMT1) at nonclassical PAMs. Full gel images of Fig 3B. Two independent transfection replicates were done, and activities assessed by T7E1 assay. (TIF) [file pbio.3002514.s008.tif]

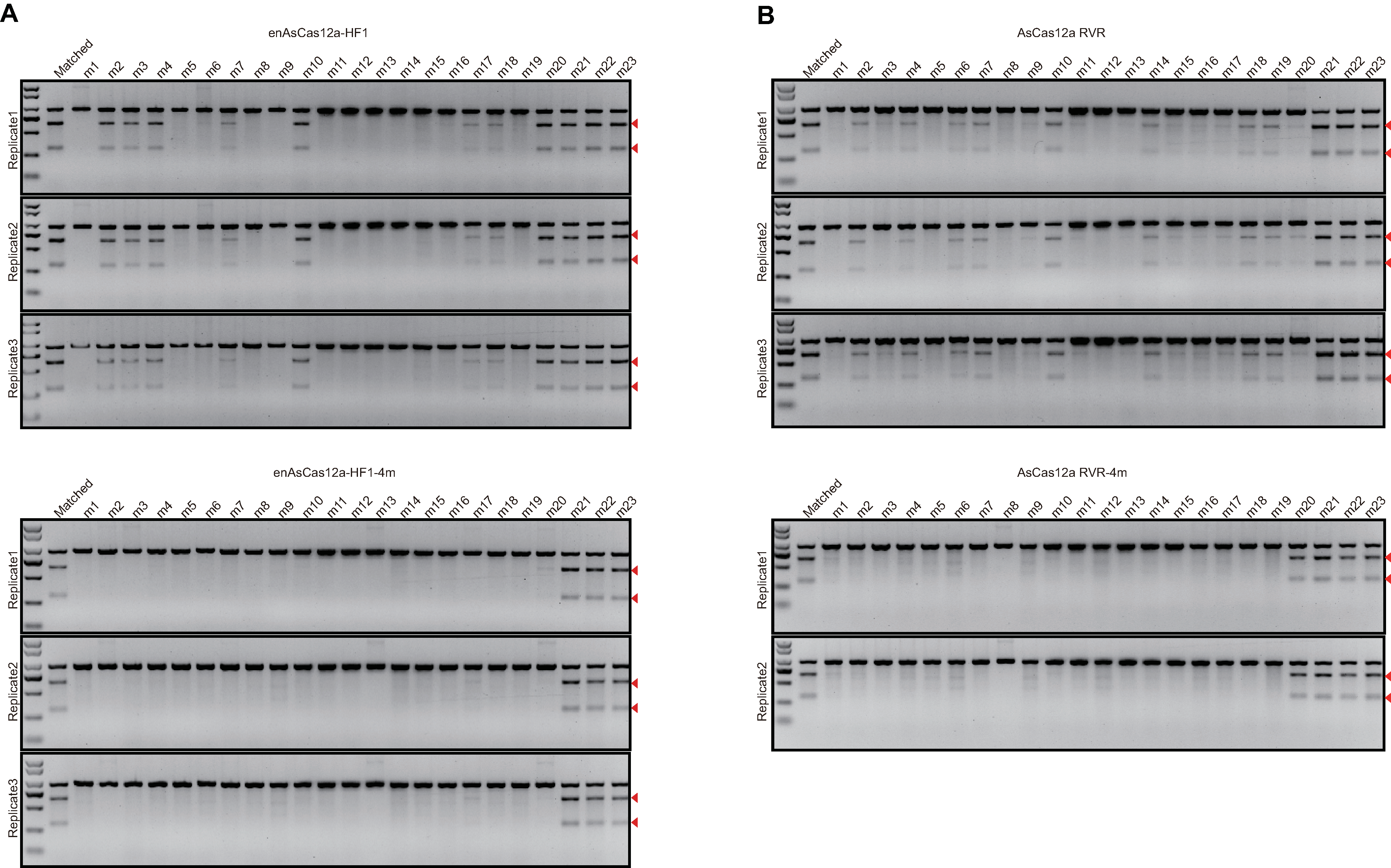

Supplement: S9 Fig — (A) Full gel images for enAsHF1 and enAsHF1-4m activities with single mismatched crRNAs toward CFTR. (B) Full gel images for AsRVR AsRVR-4m activities with single mismatched crRNAs toward CFTR. Three independent transfection replicates were done for enAsHF1, AsRVR and enAsHF1-4m and 2 independent transfection replicates were done for AsRVR-4m, activities assessed by T7E1 assay. Arrows indicates cleavage products. (TIF) [file pbio.3002514.s009.tif]

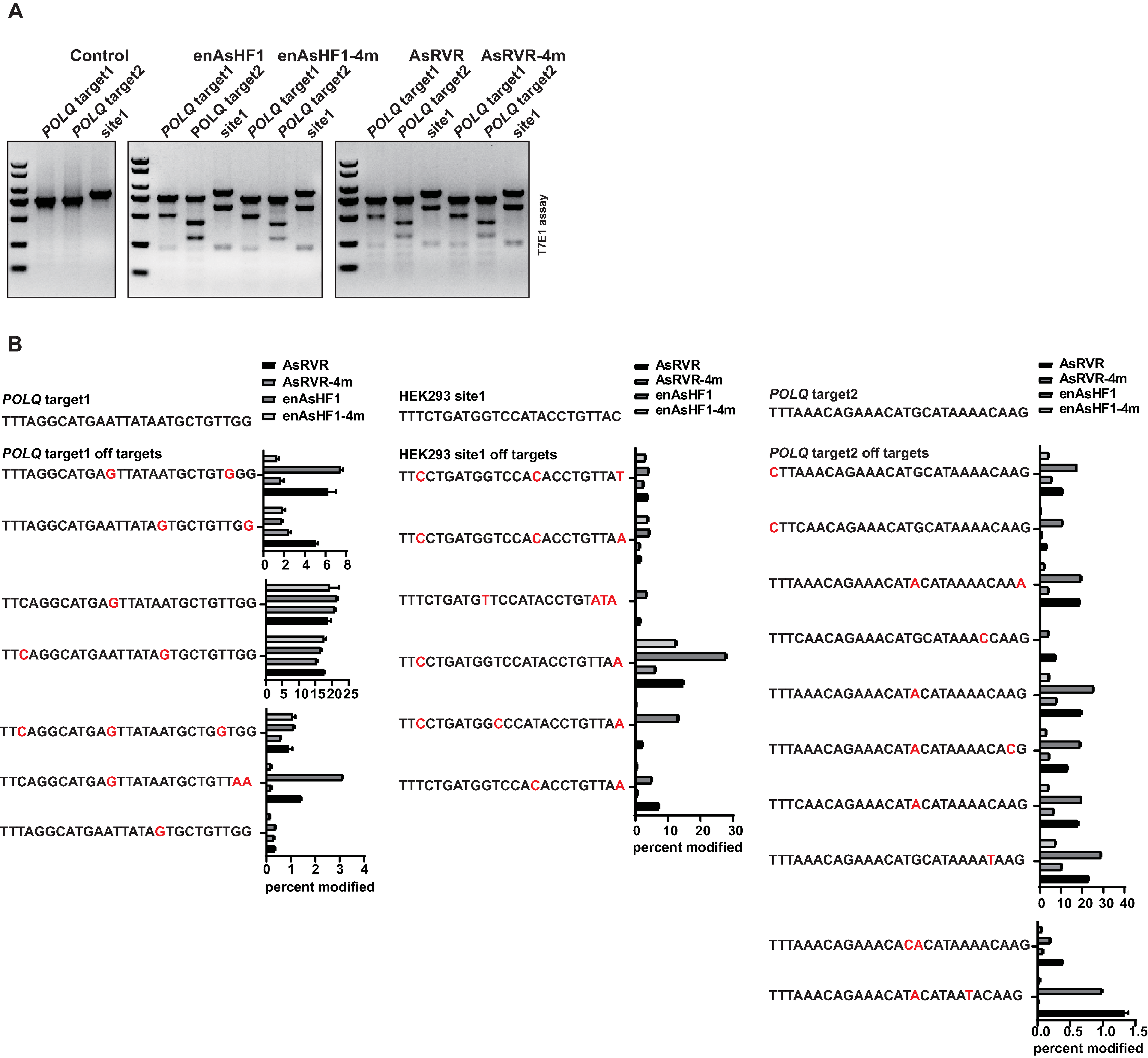

Supplement: S11 Fig — (A) On-target cleavages of these variants at 3 endogenous sites evaluated by T7 endonuclease I assay (gel images). (B) Percent modification of GUIDE-seq detected off-target sites with indel mutations for enAsHF1, AsRVR, enAsHF1-4m, and AsRVR-4m. Mismatched positions within the spacer or PAM are highlighted in red. Indel frequency assessed by deep sequencing. Error bars represent SEM for n = 2. The data underlying this figure can be found in S1 Data. (TIF) [file pbio.3002514.s011.tif]

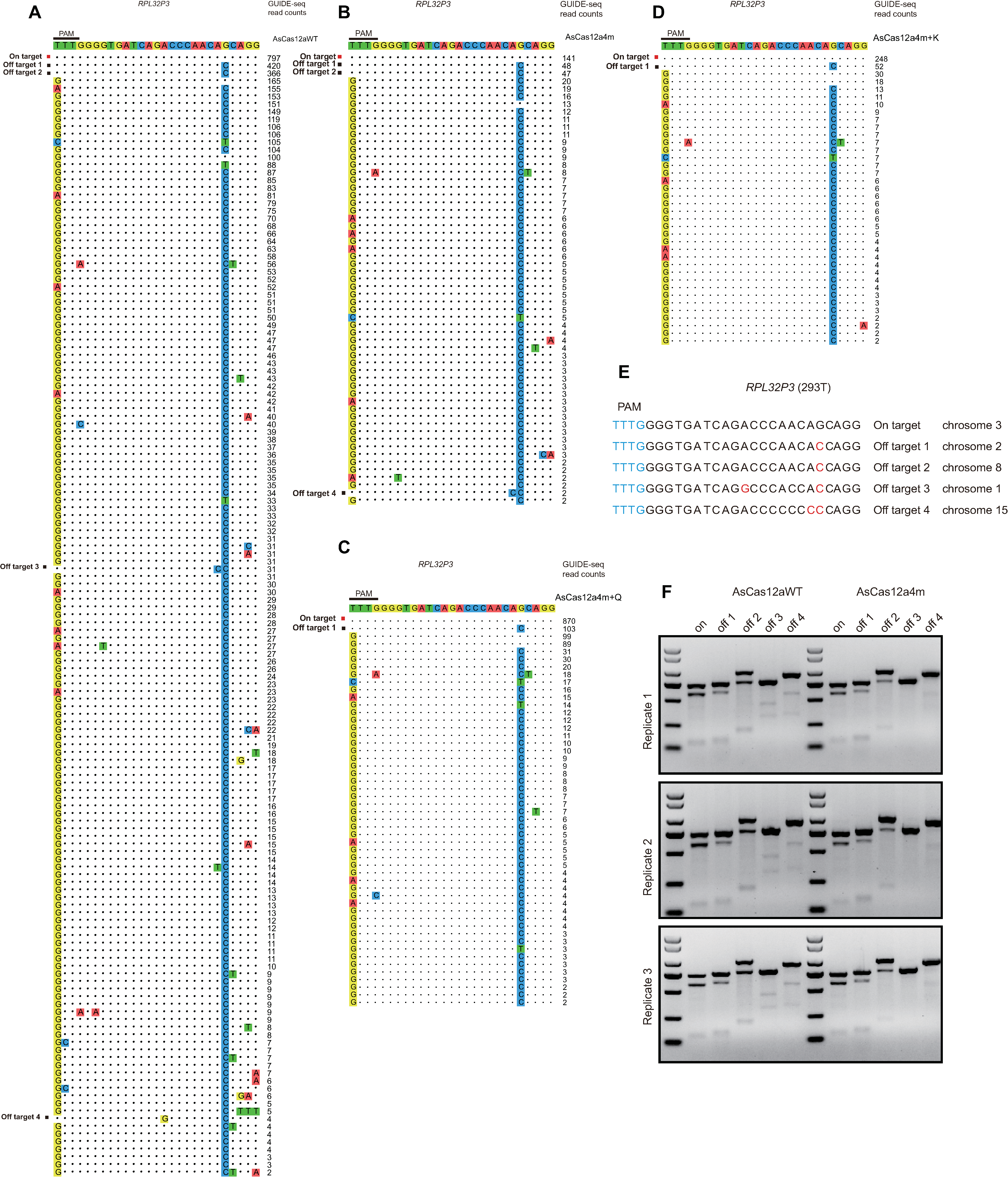

Supplement: S12 Fig — (A–D) Off-target sites for As variants with crRNA targeting RPL32P3 loci, determined using GUIDE-seq in HEK293 cells. Mismatched positions are highlighted in color, and GUIDE-seq read counts are shown to the right of the on- or off-target sequences. (E) Sequences of 4 off-target sites. (F) Full gel images for AsCas12aWT and AsCas12a4m activities toward RPL32P3 on-target site and 4 off-target sites. (TIF) [file pbio.3002514.s012.tif]

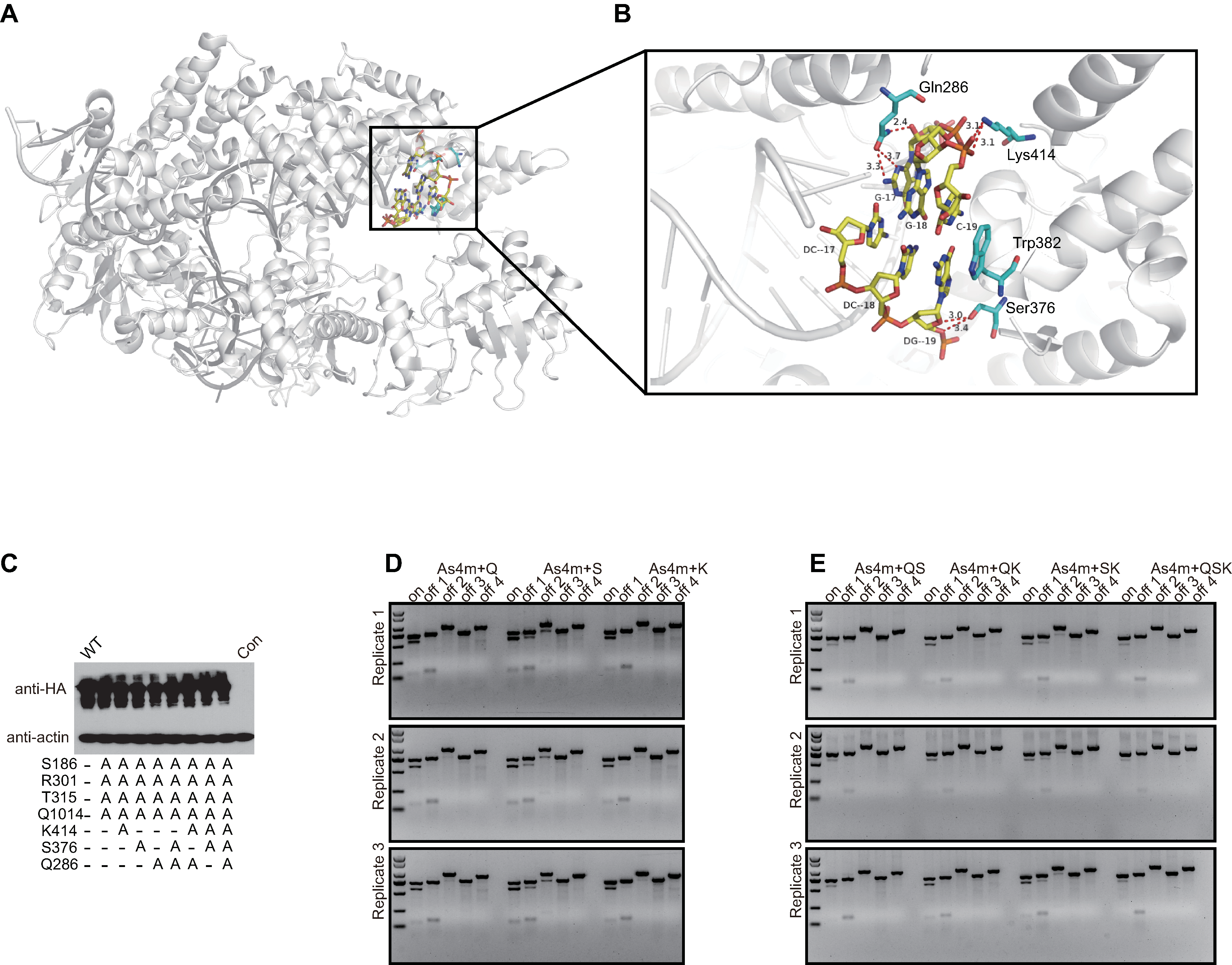

Supplement: S13 Fig — (A, B) Structural representations of AsCas12a-crRNA-DNA complex. In structural representations, amino acid residues (K414, S376, and Q286) that made direct hydrogen bonds to the phosphate backbone of the PAM-distal region (3′ end of the guide RNA and 5′ end of the target DNA). Boxes indicate regions shown in detail in B. Images generated from PDBID:5B43 (ref. [38]) visualized in PyMOL (v 1.8.6.0). (C) Expression of As variants in HEK293T cells. (D, E) Full gel images of AsCas12a4m variants cleavage profiles, 3 independent transfection replicates were done, and assessed by T7E1 assay at on-target and 4 off-targets. (TIF) [file pbio.3002514.s013.tif]

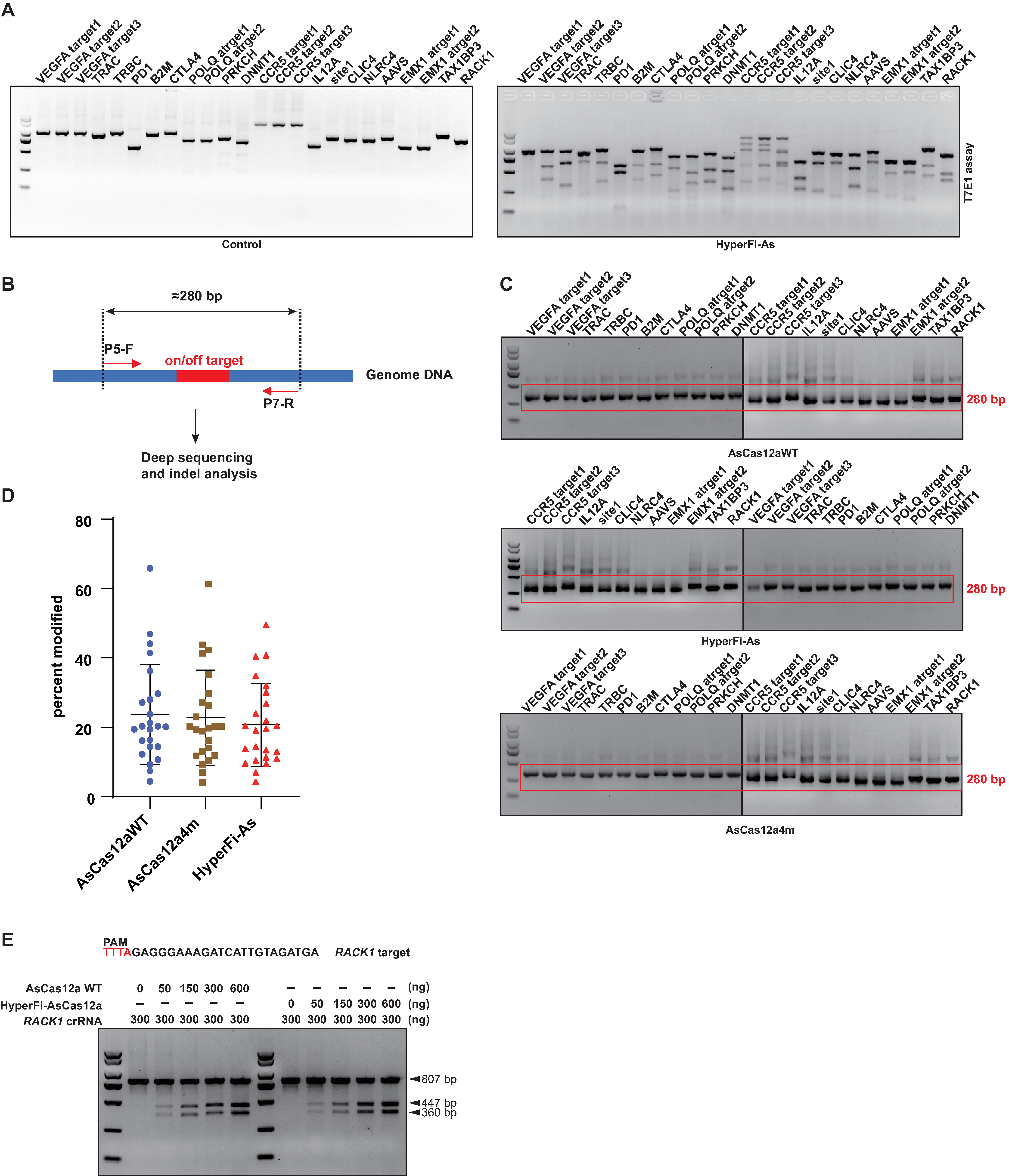

Supplement: S14 Fig — (A) Validation of HyperFi-As performance at 24 endogenous target sites with TTTV (V = A, C, or G) PAMs in HEK293T cells. (B) Schematic of the deep sequencing library constructs. Red arrows indicate genome-specific primers with Illumina P5 and P7 adapter sequences. PCR library is about 280 bp. (C) Full gel images of AsCas12aWT, AsCas12a4m, and HyperFi-As deep sequencing libraries. Red box indicated the library band of each endogenous target sites. (D) Comparison of the activity of AsCas12aWT, AsCas12a4m, and HyperFi-As in HEK293T cells. Each dot represents a target site. Indel frequency assessed by deep sequencing. The median and interquartile range are shown. (E) Evaluation of the activity of AsCas12aWT and HyperFi-AsCas12a with different amounts of plasmid. The data underlying this figure can be found in S1 Data. (TIF) [file pbio.3002514.s014.tif]
