# Supplementary material for: Engineering of Cas12a nuclease variants with enhanced genome-editing specificity
Source: PLoS Biol. 2024 Mar 14;22(3):e3002514. doi: 10.1371/journal.pbio.3002514 (PMC10965058; doi:10.1371/journal.pbio.3002514)
Supplement: S1 Table — (DOCX) [file pbio.3002514.s023.docx]

**S1 Table.** List of sequences used in study

| Target ID | PAM | Guide sequence (5' to 3') |  | Assay |
| --- | --- | --- | --- | --- |
|  |  |  |  |  |
| EGFP | TTTA | CGTCGCCGTCCAGCTCGACCAGG | Fully match | EGFP disruption |
| EGFP | TTTA | CGTCGCCGTCCAGCTCGACCACC | m23&22 | EGFP disruption |
| EGFP | TTTA | CGTCGCCGTCCAGCTCGACCTGC | m23&21 | EGFP disruption |
| EGFP | TTTA | CGTCGCCGTCCAGCTCGACGAGC | m23&20 | EGFP disruption |
| EGFP | TTTA | CGTCGCCGTCCAGCTCGAGCAGC | m23&19 | EGFP disruption |
| EGFP | TTTA | CGTCGCCGTCCAGCTCGTCCAGC | m23&18 | EGFP disruption |
| EGFP | TTTA | CGTCGCCGTCCAGCTCCACCAGC | m23&17 | EGFP disruption |
| EGFP | TTTA | CGTCGCCGTCCAGCTGGACCAGC | m23&16 | EGFP disruption |
| EGFP | TTTA | CGTCGCCGTCCAGCACGACCAGC | m23&15 | EGFP disruption |
| EGFP | TTTA | CGTCGCCGTCCAGGTCGACCAGC | m23&14 | EGFP disruption |
| EGFP | TTTA | CGTCGCCGTCCAGCTCGACCTCG | m22&21 | EGFP disruption |
| EGFP | TTTA | CGTCGCCGTCCAGCTCGACGACG | m22&20 | EGFP disruption |
| EGFP | TTTA | CGTCGCCGTCCAGCTCGAGCACG | m22&19 | EGFP disruption |
| EGFP | TTTA | CGTCGCCGTCCAGCTCGTCCACG | m22&18 | EGFP disruption |
| EGFP | TTTA | CGTCGCCGTCCAGCTCCACCACG | m22&17 | EGFP disruption |
| EGFP | TTTA | CGTCGCCGTCCAGCTGGACCACG | m22&16 | EGFP disruption |
| EGFP | TTTA | CGTCGCCGTCCAGCACGACCACG | m22&15 | EGFP disruption |
| EGFP | TTTA | CGTCGCCGTCCAGGTCGACCACG | m22&14 | EGFP disruption |
| EGFP | TTTA | CGTCGCCGTCCAGCTCGACGTGG | m21&20 | EGFP disruption |
| EGFP | TTTA | CGTCGCCGTCCAGCTCGAGCTGG | m21&19 | EGFP disruption |
| EGFP | TTTA | CGTCGCCGTCCAGCTCGTCCTGG | m21&18 | EGFP disruption |
| EGFP | TTTA | CGTCGCCGTCCAGCTCCACCTGG | m21&17 | EGFP disruption |
| EGFP | TTTA | CGTCGCCGTCCAGCTGGACCTGG | m21&16 | EGFP disruption |
| EGFP | TTTA | CGTCGCCGTCCAGCACGACCTGG | m21&15 | EGFP disruption |
| EGFP | TTTA | CGTCGCCGTCCAGGTCGACCTGG | m21&14 | EGFP disruption |
| EGFP | TTTA | CGTCGCCGTCCAGCTCGAGGAGG | m20&19 | EGFP disruption |
| EGFP | TTTA | CGTCGCCGTCCAGCTCGTCGAGG | m20&18 | EGFP disruption |
| EGFP | TTTA | CGTCGCCGTCCAGCTCCACGAGG | m20&17 | EGFP disruption |
| EGFP | TTTA | CGTCGCCGTCCAGCTGGACGAGG | m20&16 | EGFP disruption |
| EGFP | TTTA | CGTCGCCGTCCAGCACGACGAGG | m20&15 | EGFP disruption |
| EGFP | TTTA | CGTCGCCGTCCAGGTCGACGAGG | m20&14 | EGFP disruption |
| EGFP | TTTA | CGTCGCCGTCCAGCTCGTGCAGG | m19&18 | EGFP disruption |
| EGFP | TTTA | CGTCGCCGTCCAGCTCCAGCAGG | m19&17 | EGFP disruption |
| EGFP | TTTA | CGTCGCCGTCCAGCTGGAGCAGG | m19&16 | EGFP disruption |
| EGFP | TTTA | CGTCGCCGTCCAGCACGAGCAGG | m19&15 | EGFP disruption |
| EGFP | TTTA | CGTCGCCGTCCAGGTCGAGCAGG | m19&14 | EGFP disruption |
| EGFP | TTTA | CGTCGCCGTCCAGCTCCTCCAGG | m18&17 | EGFP disruption |
| EGFP | TTTA | CGTCGCCGTCCAGCTGGTCCAGG | m18&16 | EGFP disruption |
| EGFP | TTTA | CGTCGCCGTCCAGCACGTCCAGG | m18&15 | EGFP disruption |
| EGFP | TTTA | CGTCGCCGTCCAGGTCGTCCAGG | m18&14 | EGFP disruption |
| EGFP | TTTA | CGTCGCCGTCCAGCTGCACCAGG | m17&16 | EGFP disruption |
| EGFP | TTTA | CGTCGCCGTCCAGCACCACCAGG | m17&15 | EGFP disruption |
| EGFP | TTTA | CGTCGCCGTCCAGGTCCACCAGG | m17&14 | EGFP disruption |
| EGFP | TTTA | CGTCGCCGTCCAGCAGGACCAGG | m16&15 | EGFP disruption |
| EGFP | TTTA | CGTCGCCGTCCAGGTGGACCAGG | m16&14 | EGFP disruption |
| EGFP | TTTA | CGTCGCCGTCCAGGACGACCAGG | m15&14 | EGFP disruption |
| CFTR | TTTA | GAGAGAAGGCTGTCCTTAGTACC | Fully match | T7EI |
| CFTR | TTTA | CAGAGAAGGCTGTCCTTAGTACC | m1 | T7EI |
| CFTR | TTTA | GTGAGAAGGCTGTCCTTAGTACC | m2 | T7EI |
| CFTR | TTTA | GACAGAAGGCTGTCCTTAGTACC | m3 | T7EI |
| CFTR | TTTA | GAGTGAAGGCTGTCCTTAGTACC | m4 | T7EI |
| CFTR | TTTA | GAGACAAGGCTGTCCTTAGTACC | m5 | T7EI |
| CFTR | TTTA | GAGAGTAGGCTGTCCTTAGTACC | m6 | T7EI |
| CFTR | TTTA | GAGAGATGGCTGTCCTTAGTACC | m7 | T7EI |
| CFTR | TTTA | GAGAGAACGCTGTCCTTAGTACC | m8 | T7EI |
| CFTR | TTTA | GAGAGAAGCCTGTCCTTAGTACC | m9 | T7EI |
| CFTR | TTTA | GAGAGAAGGGTGTCCTTAGTACC | m10 | T7EI |
| CFTR | TTTA | GAGAGAAGGCAGTCCTTAGTACC | m11 | T7EI |
| CFTR | TTTA | GAGAGAAGGCTCTCCTTAGTACC | m12 | T7EI |
| CFTR | TTTA | GAGAGAAGGCTGACCTTAGTACC | m13 | T7EI |
| CFTR | TTTA | GAGAGAAGGCTGTGCTTAGTACC | m14 | T7EI |
| CFTR | TTTA | GAGAGAAGGCTGTCGTTAGTACC | m15 | T7EI |
| CFTR | TTTA | GAGAGAAGGCTGTCCATAGTACC | m16 | T7EI |
| CFTR | TTTA | GAGAGAAGGCTGTCCTAAGTACC | m17 | T7EI |
| CFTR | TTTA | GAGAGAAGGCTGTCCTTTGTACC | m18 | T7EI |
| CFTR | TTTA | GAGAGAAGGCTGTCCTTACTACC | m19 | T7EI |
| CFTR | TTTA | GAGAGAAGGCTGTCCTTAGAACC | m20 | T7EI |
| CFTR | TTTA | GAGAGAAGGCTGTCCTTAGTTCC | m21 | T7EI |
| CFTR | TTTA | GAGAGAAGGCTGTCCTTAGTAGC | m22 | T7EI |
| CFTR | TTTA | GAGAGAAGGCTGTCCTTAGTACG | m23 | T7EI |
| CFTR | TTTA | CTGAGAAGGCTGTCCTTAGTACC | m1&2 | T7EI |
| CFTR | TTTA | GACTGAAGGCTGTCCTTAGTACC | m3&4 | T7EI |
| CFTR | TTTA | GAGACTAGGCTGTCCTTAGTACC | m5&6 | T7EI |
| CFTR | TTTA | GAGAGATCGCTGTCCTTAGTACC | m7&8 | T7EI |
| CFTR | TTTA | GAGAGAAGCGTGTCCTTAGTACC | m9&10 | T7EI |
| CFTR | TTTA | GAGAGAAGGCACTCCTTAGTACC | m11&12 | T7EI |
| CFTR | TTTA | GAGAGAAGGCTGAGCTTAGTACC | m13&14 | T7EI |
| CFTR | TTTA | GAGAGAAGGCTGTCGATAGTACC | m15&16 | T7EI |
| CFTR | TTTA | GAGAGAAGGCTGTCCTATGTACC | m17&18 | T7EI |
| CFTR | TTTA | GAGAGAAGGCTGTCCTTACAACC | m19&20 | T7EI |
| CFTR | TTTA | GAGAGAAGGCTGTCCTTAGTTGC | m21&22 | T7EI |
| B2M | TTTA | CTCACGTCATCCAGCAGAGAATG | Fully match | T7EI |
| B2M | TTTA | gTCACGTCATCCAGCAGAGAATG | m1 | T7EI |
| B2M | TTTA | CaCACGTCATCCAGCAGAGAATG | m2 | T7EI |
| B2M | TTTA | CTgACGTCATCCAGCAGAGAATG | m3 | T7EI |
| B2M | TTTA | CTCtCGTCATCCAGCAGAGAATG | m4 | T7EI |
| B2M | TTTA | CTCAgGTCATCCAGCAGAGAATG | m5 | T7EI |
| B2M | TTTA | CTCACcTCATCCAGCAGAGAATG | m6 | T7EI |
| B2M | TTTA | CTCACGaCATCCAGCAGAGAATG | m7 | T7EI |
| B2M | TTTA | CTCACGTgATCCAGCAGAGAATG | m8 | T7EI |
| B2M | TTTA | CTCACGTCtTCCAGCAGAGAATG | m9 | T7EI |
| B2M | TTTA | CTCACGTCAaCCAGCAGAGAATG | m10 | T7EI |
| B2M | TTTA | CTCACGTCATgCAGCAGAGAATG | m11 | T7EI |
| B2M | TTTA | CTCACGTCATCgAGCAGAGAATG | m12 | T7EI |
| B2M | TTTA | CTCACGTCATCCtGCAGAGAATG | m13 | T7EI |
| B2M | TTTA | CTCACGTCATCCAcCAGAGAATG | m14 | T7EI |
| B2M | TTTA | CTCACGTCATCCAGgAGAGAATG | m15 | T7EI |
| B2M | TTTA | CTCACGTCATCCAGCtGAGAATG | m16 | T7EI |
| B2M | TTTA | CTCACGTCATCCAGCAcAGAATG | m17 | T7EI |
| B2M | TTTA | CTCACGTCATCCAGCAGtGAATG | m18 | T7EI |
| B2M | TTTA | CTCACGTCATCCAGCAGAcAATG | m19 | T7EI |
| B2M | TTTA | CTCACGTCATCCAGCAGAGtATG | m20 | T7EI |
| B2M | TTTA | CTCACGTCATCCAGCAGAGAtTG | m21 | T7EI |
| B2M | TTTA | CTCACGTCATCCAGCAGAGAAaG | m22 | T7EI |
| B2M | TTTA | CTCACGTCATCCAGCAGAGAATc | m23 | T7EI |
| B2M | TTTA | gaCACGTCATCCAGCAGAGAATG | m1&2 | T7EI |
| B2M | TTTA | CTgtCGTCATCCAGCAGAGAATG | m3&4 | T7EI |
| B2M | TTTA | CTCAgcTCATCCAGCAGAGAATG | m5&6 | T7EI |
| B2M | TTTA | CTCACGagATCCAGCAGAGAATG | m7&8 | T7EI |
| B2M | TTTA | CTCACGTCtaCCAGCAGAGAATG | m9&10 | T7EI |
| B2M | TTTA | CTCACGTCATggAGCAGAGAATG | m11&12 | T7EI |
| B2M | TTTA | CTCACGTCATCCtcCAGAGAATG | m13&14 | T7EI |
| B2M | TTTA | CTCACGTCATCCAGgtGAGAATG | m15&16 | T7EI |
| B2M | TTTA | CTCACGTCATCCAGCActGAATG | m17&18 | T7EI |
| B2M | TTTA | CTCACGTCATCCAGCAGActATG | m19&20 | T7EI |
| B2M | TTTA | CTCACGTCATCCAGCAGAGAtaG | m21&22 | T7EI |
| AAVS | TTTG | CTTACGATGGAGCCAGAGAGGAT |  | T7EI |
| CCR5 target1 | TTTC | CTGCTCCCCAGTGGATCGGGTGT |  | T7EI |
| CCR5 target2 | TTTG | GCATCTGTTTAAAGTAGATTAGA |  | T7EI |
| DNMT1 target1 | TTTC | CCTCACTCCTGCTCGGTGAATTT |  | T7EI |
| DNMT1 target2 | TTTC | CTGATGGTCCATGTCTGTTACTC |  | T7EI |
| DNMT1 target3 | TTTG | GCTCAGCAGGCACCTGCCTCAGC |  | T7EI |
| FANCF target1 | TTTG | GTCGGCATGGCCCCATTCGCACG |  | T7EI |
| FANCF target2 | TTTG | GGCGGGGTCCAGTTCCGGGATTA |  | T7EI |
| HBB target1 | TTTG | GGGATCTGTCCACTCCTGATGCT |  | T7EI |
| HBB target2 | TTTG | TTGCCATGAGCCTTCACCTTAGG |  | T7EI |
| VEGFA target1 | TTTG | CTAGGAATATTGAAGGGGGCAGG |  | T7EI |
| VEGFA target2 | TTTG | GGAGGTCAGAAATAGGGGGTCCA |  | T7EI |
| VEGFA target3 | TTTG | GGACTGGAGTTGCTTCATGTACA |  | T7EI |
| B2M | TTTA | CTCACGTCATCCAGCAGAGAATG |  | T7EI |
| CTLA4 | TTTC | AGCGGCACAAGGCTCAGCTGAAC |  | T7EI |
| PD1 | TTTA | GCACGAAGCTCTCCGATGTGTTG |  | T7EI |
| RPL32P3 | TTTG | GGGTGATCAGACCCAACAGCAGG |  | T7EI |
| CFTR | TTCC | ATTGTGCAAAAGACTCCCTTACA |  | T7EI |
| CFTR | TTCA | ATCCTAACTGAGACCTTACACCG |  | T7EI |
| CTLA4 | TATG | GGAGCGGTGTTCAGGTCTTC |  | T7EI |
| CTLA4 | TATG | TATTACACATGTGCACACAC |  | T7EI |
| DNMT1 | CTTA | GCACTCTGCCACTTATTGGGTCA |  | T7EI |
| DNMT1 | CTTA | TTGGGTCAGCTGTTAACATCAGT |  | T7EI |
| RPL32P3 off1 | TTTG | GGGTGATCAGACCCAACACCAGG |  | T7EI |
| RPL32P3 off2 | TTTG | GGGTGATCAGACCCAACACCAGG |  | T7EI |
| RPL32P3 off3 | TTTG | GGGTGATCAGGCCCAACACCAGG |  | T7EI |
| RPL32P3 off4 | TTTG | GGGTGATCAGACCCAACCCCAGG |  | T7EI |
| PD1 | TTTA | GCACGAAGCTCTCCGATGTGTTG |  | GUIDE seq |
| B2M | TTTA | CTCACGTCATCCAGCAGAGAATG |  | GUIDE seq |
| HPRT | TTTG | CTGACCTGCTGGATTACATCAAA |  | GUIDE seq |
| CLIC4 | TTTA | CCCTGGCTACCTCCCCTACC |  | GUIDE seq |
| NLRC4 | TTTA | GAGGGAGACACAAGTTGATA |  | GUIDE seq |
| DNMT1 | TTTG | GCTCAGCAGGCACCTGCCTC |  | GUIDE seq |
| IL12A | TTTA | GGATGCCACTAAAAGGGAAA |  | GUIDE seq |
| HEK293 site1 | TTTC | TGATGGTCCATACCTGTTACACT |  | GUIDE seq |
| POLQ target1 | TTTA | GGCATGAATTATAATGCTGTTGG |  | GUIDE seq |
| POLQ target2 | TTTA | AACAGAAACATGCATAAAACAAG |  | GUIDE seq |
| SIPRa | TTTA | AAAAGAGAGAGAGAGGGGGGAAA |  | GUIDE seq |
| PPP1R13L | TTTA | TTGAGCAGTTAGTATGTGCCTGG |  | GUIDE seq |
| HEK293 site2 | TTTC | TTTATCCAGTTTACCATTCATGG |  | GUIDE seq |
| HEK293 site3 | TTTA | ATTGGACTCACAGTGCTACATGG |  | GUIDE seq |
| HEK293 site4 | TTTA | GCAGGCTTGGAGGACTGTGTTGG |  | GUIDE seq |
| HEK293 site5 | TTTG | TGCAACATGGTTGGAGCTGGAGG |  | GUIDE seq |
| HEK293 site6 | TTTC | AATTCTCTCATCCATAAAATGGG |  | GUIDE seq |
