# Supplementary material for: Engineering of Cas12a nuclease variants with enhanced genome-editing specificity
Source: PLoS Biol. 2024 Mar 14;22(3):e3002514. doi: 10.1371/journal.pbio.3002514 (PMC10965058; doi:10.1371/journal.pbio.3002514)

Extended Data Fig. 1: Uncropped gels for Fig 1C and S1F Fig.

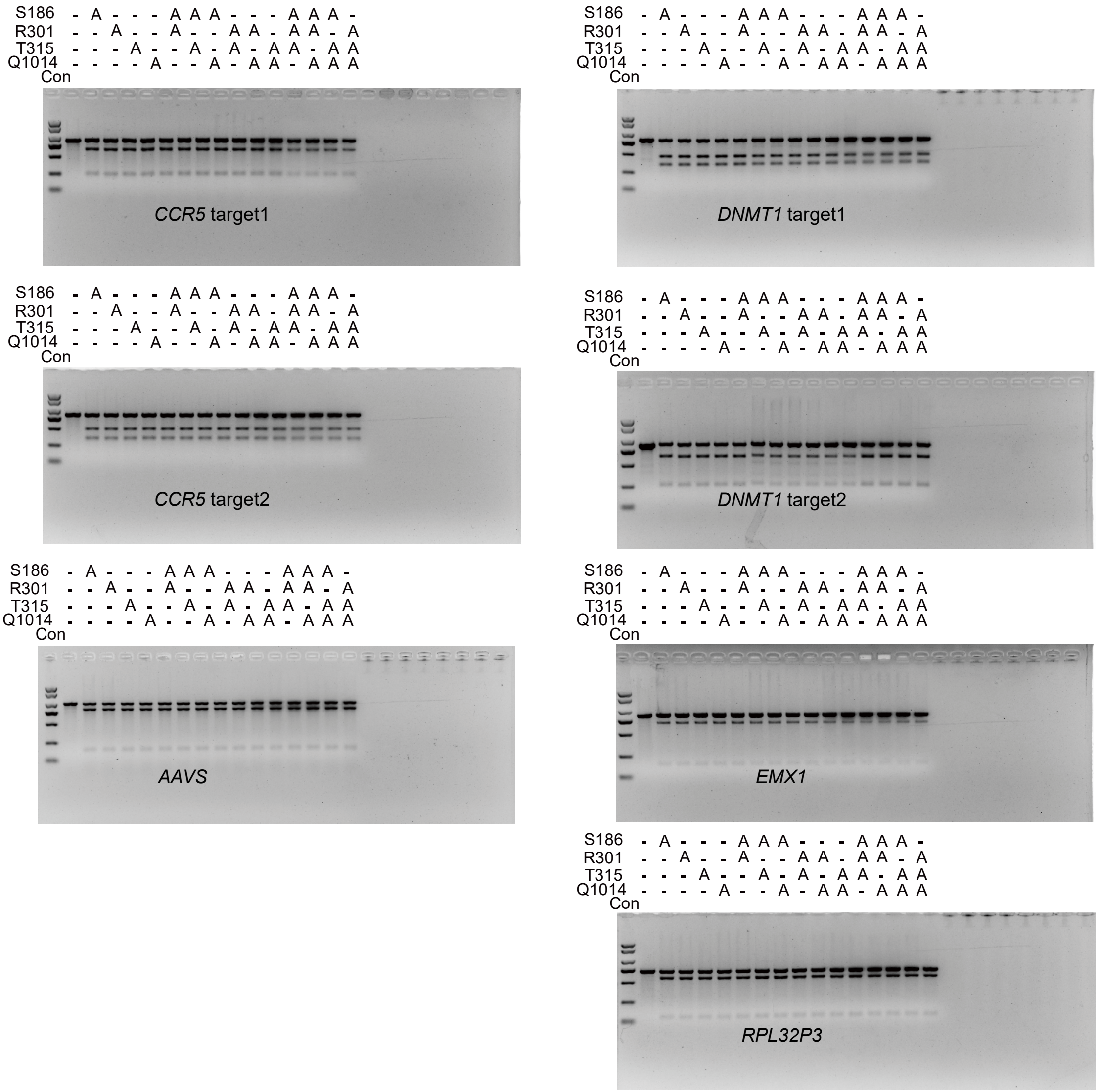

Extended Data Fig. 2: Uncropped gels for Fig 1D and 1E and S2 Fig.

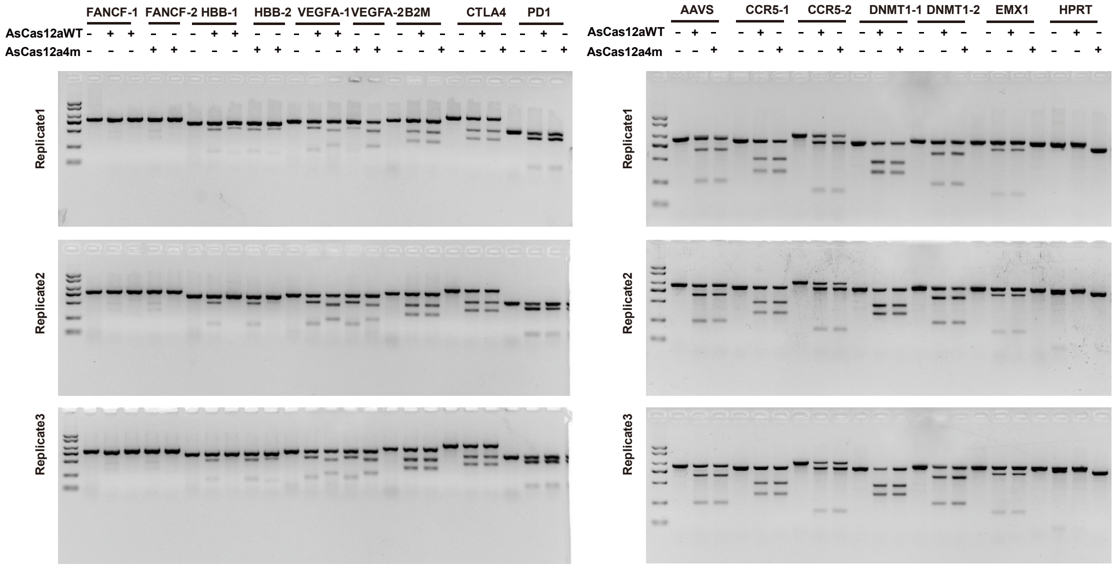

Extended Data Fig. 3: Uncropped gels for Fig 2A and S3A Fig.

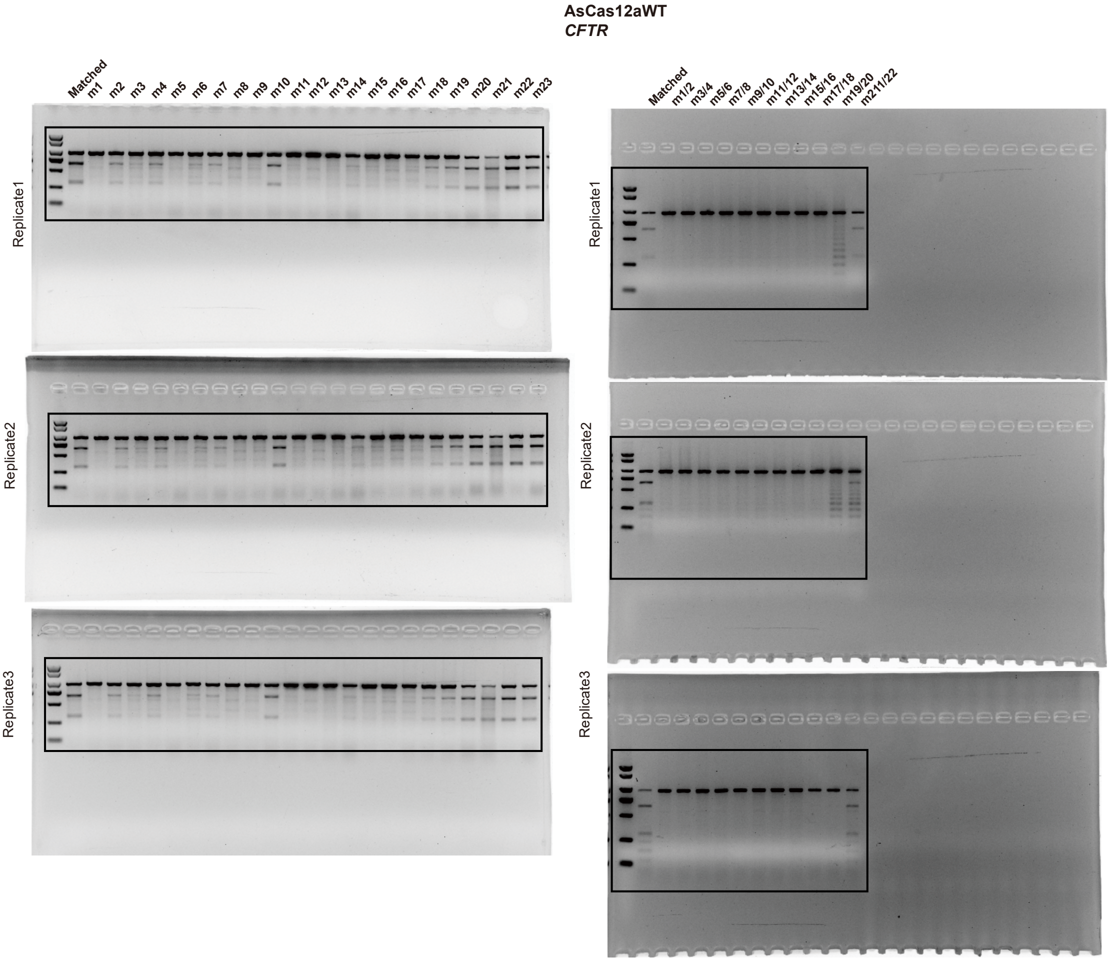

Extended Data Fig. 4: Uncropped gels for Fig 2A and S3B Fig.

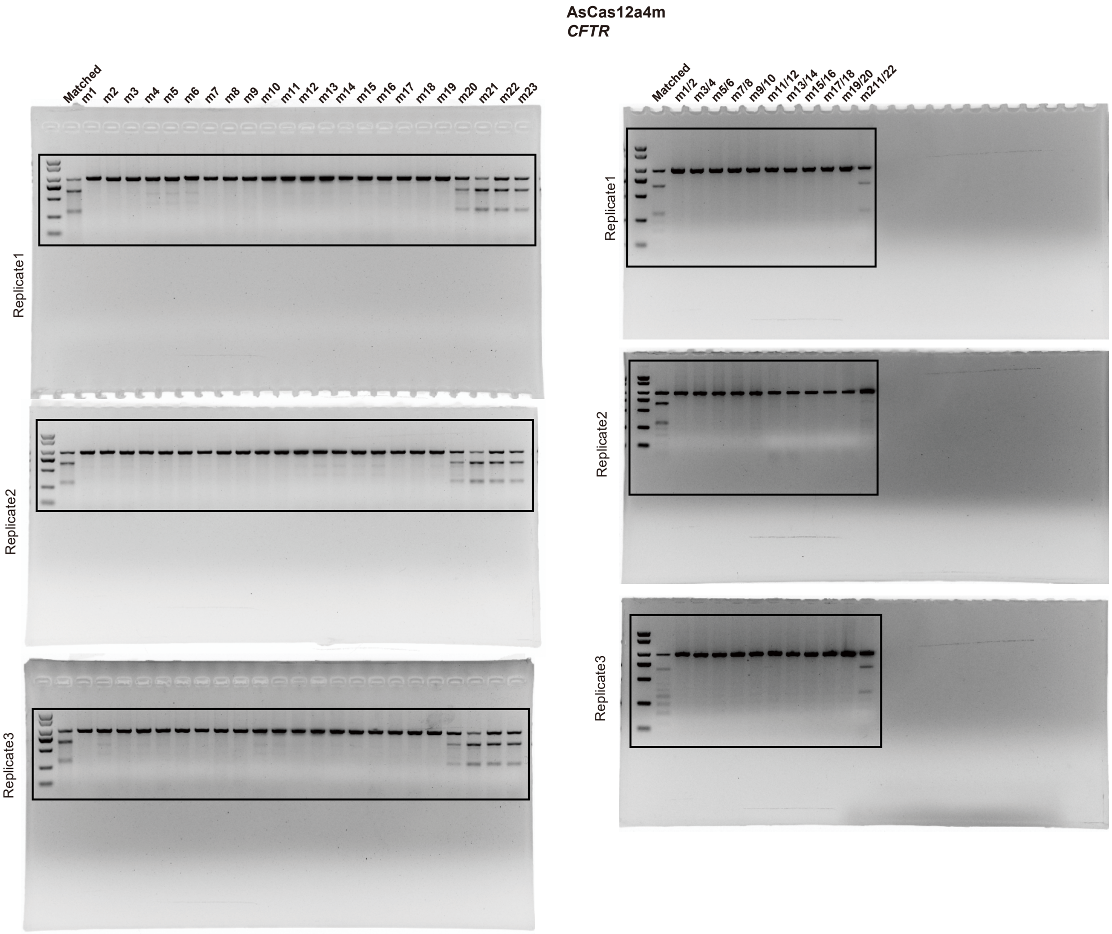

Extended Data Fig. 5: Uncropped gels for Fig 2B and S4A Fig.

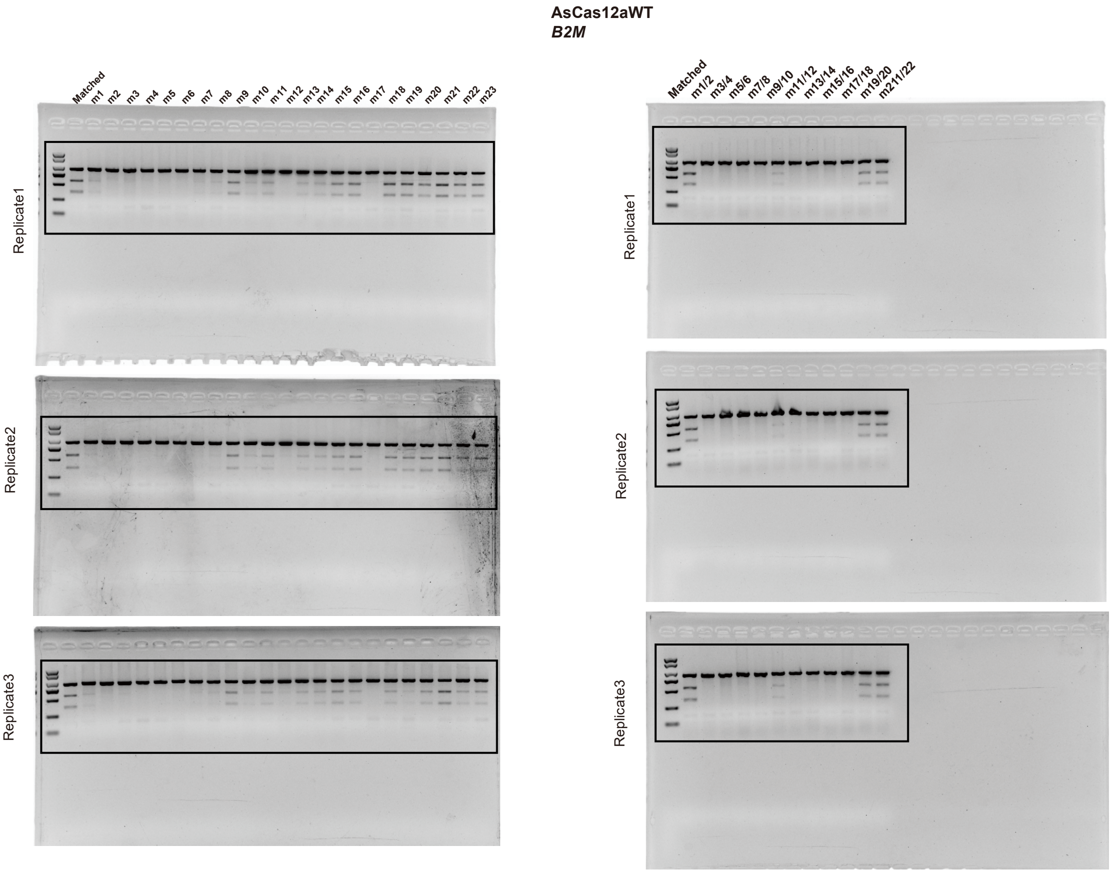

Extended Data Fig. 6: Uncropped gels for Fig 2B and S4B Fig.

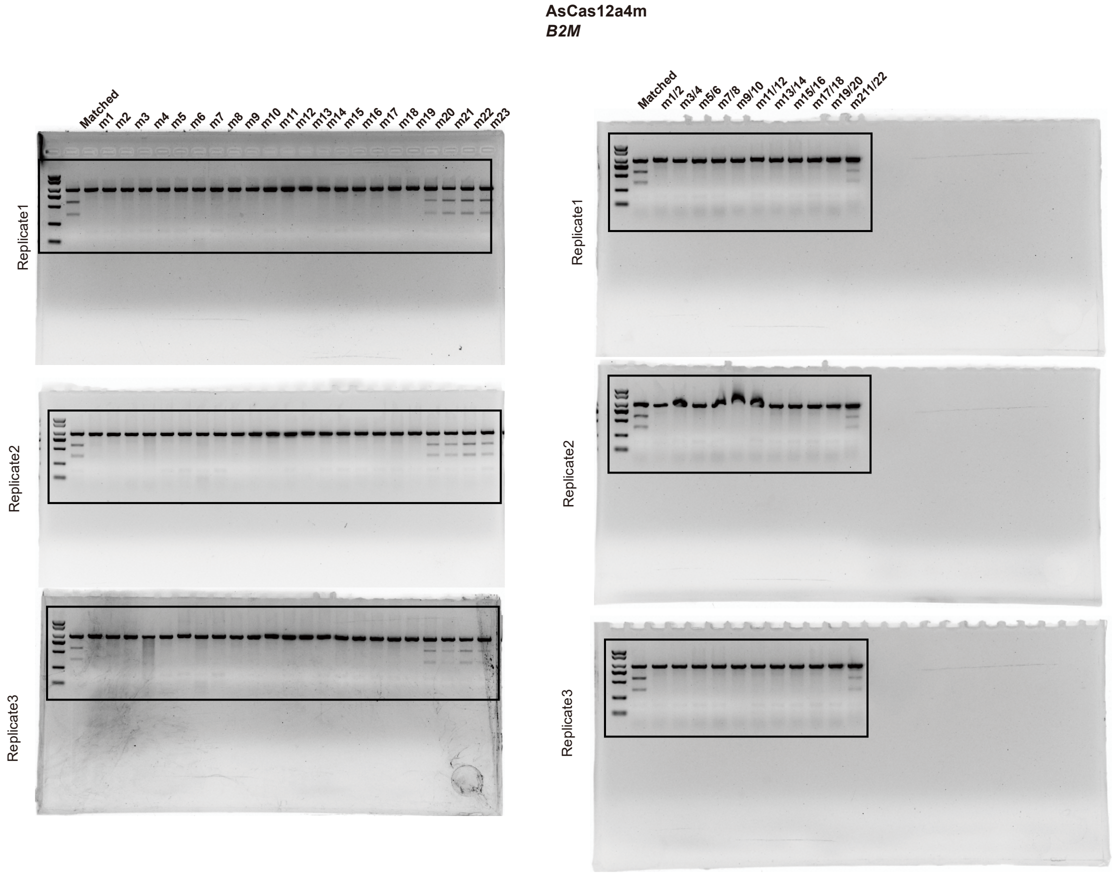

Extended Data Fig. 7: Uncropped gels for S5B Fig.

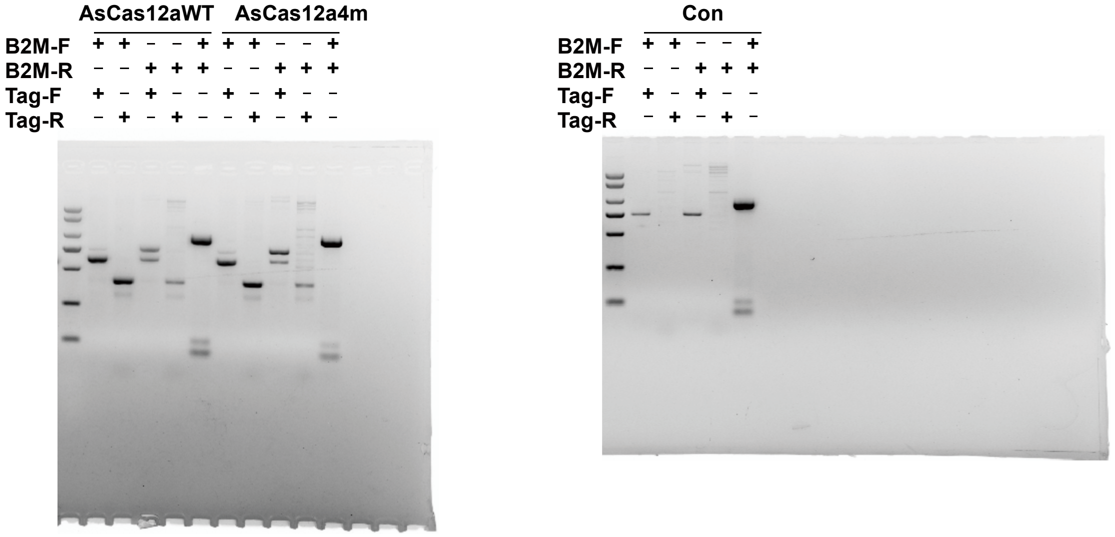

Extended Data Fig. 8: Uncropped gels for Fig 3A and S8A Fig.

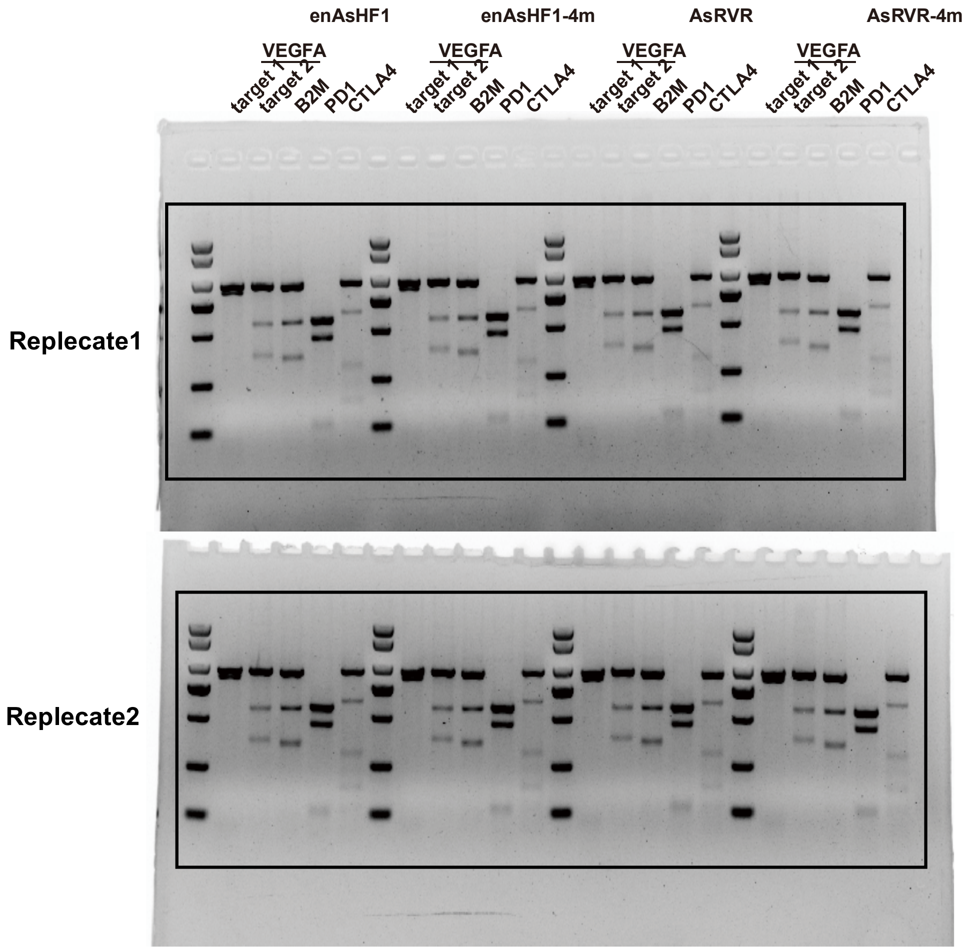

Extended Data Fig. 9: Uncropped gels for Fig 3B and S8B Fig.

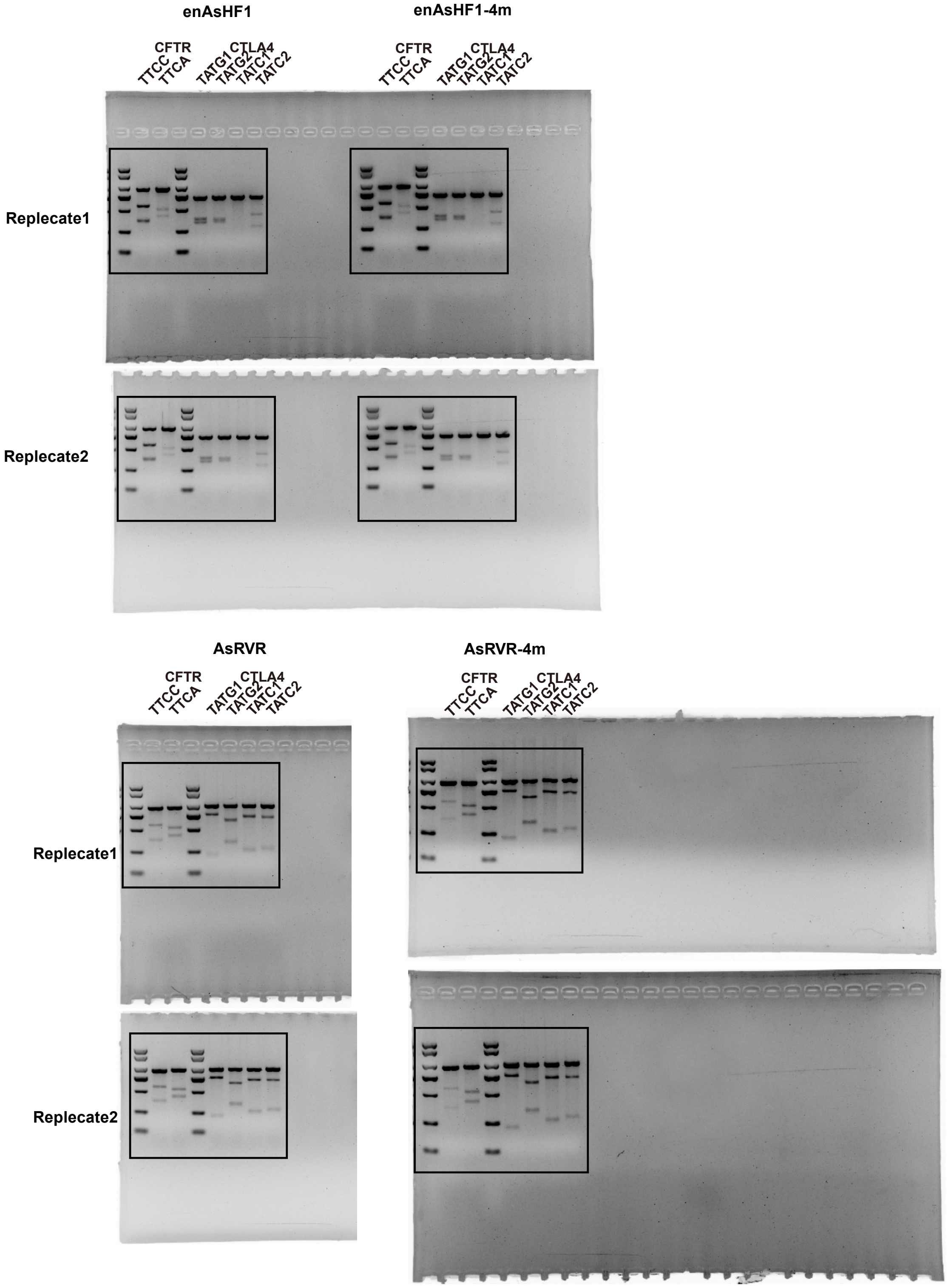

Extended Data Fig. 10: Uncropped gels for Fig 3D and S9A Fig.

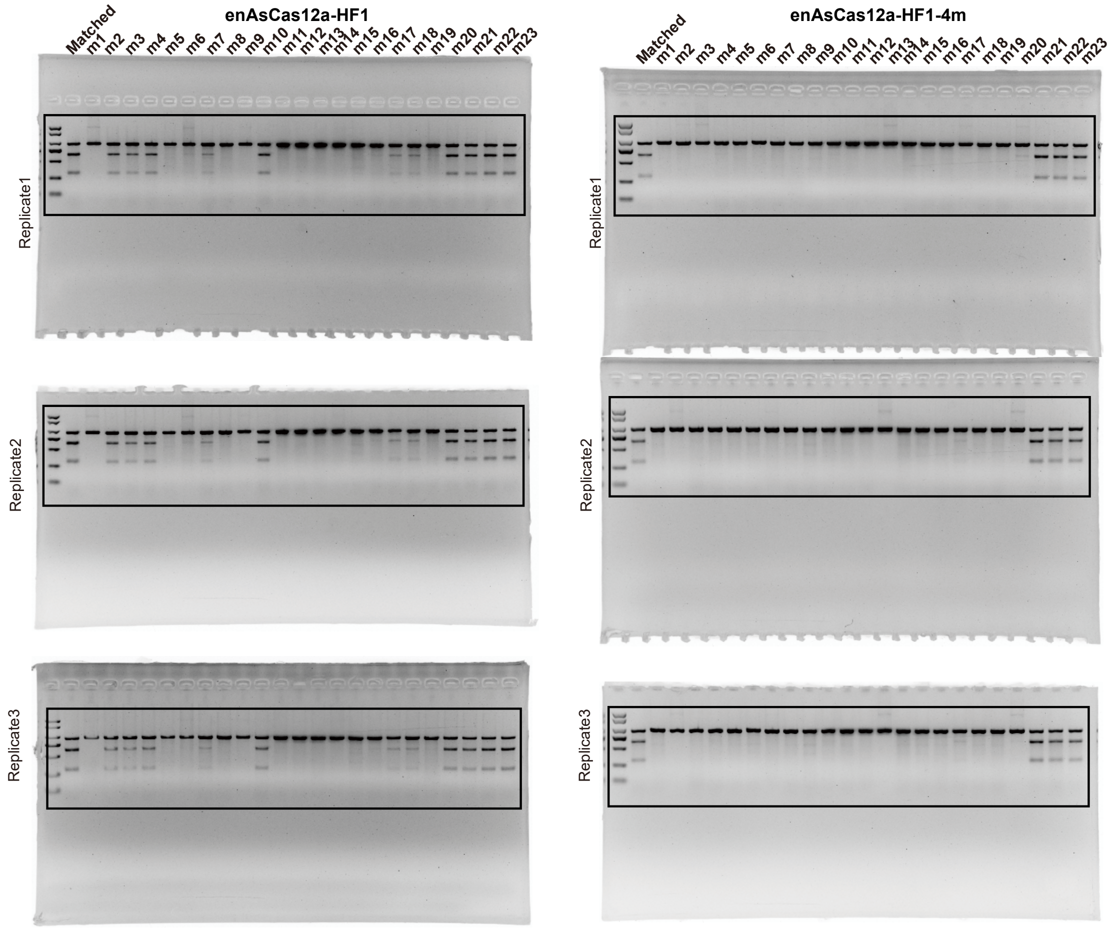

Extended Data Fig. 11: Uncropped gels for Fig 3C and S9B Fig.

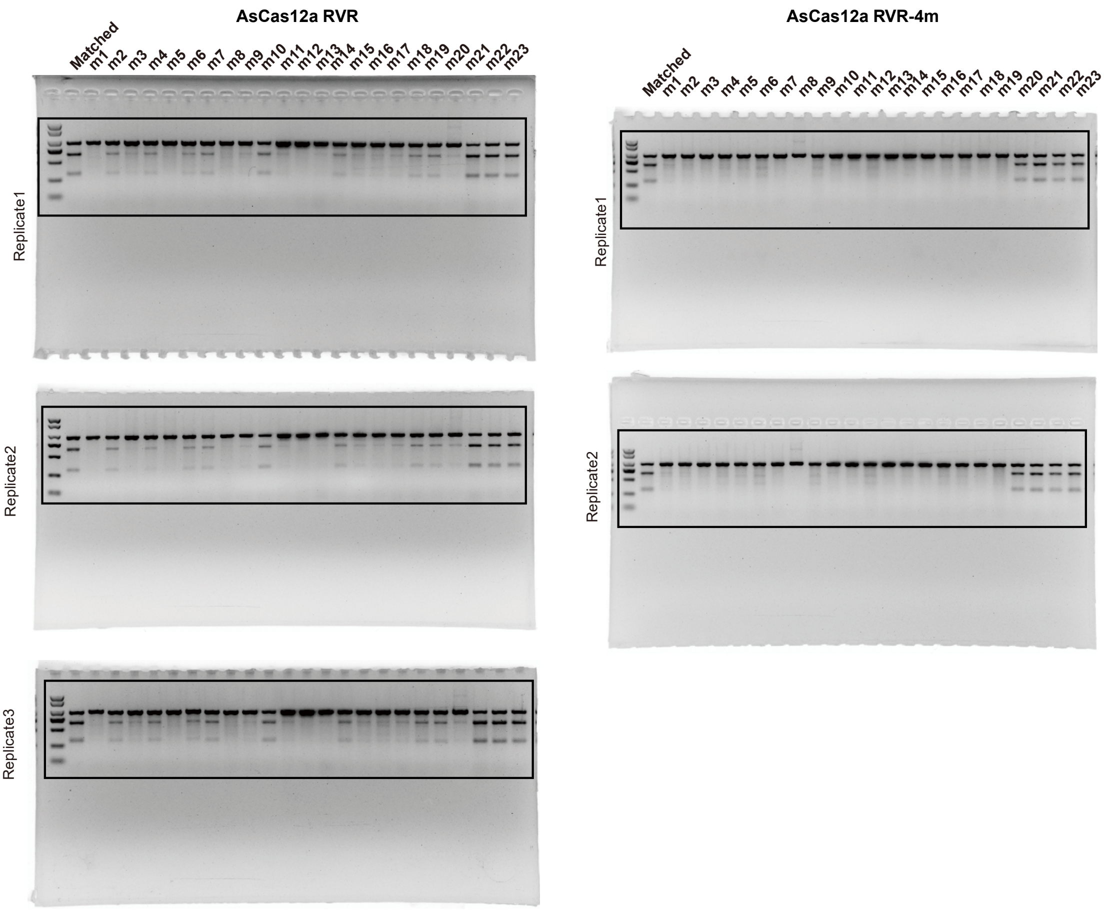

Extended Data Fig. 12: Uncropped gels for S11A Fig.

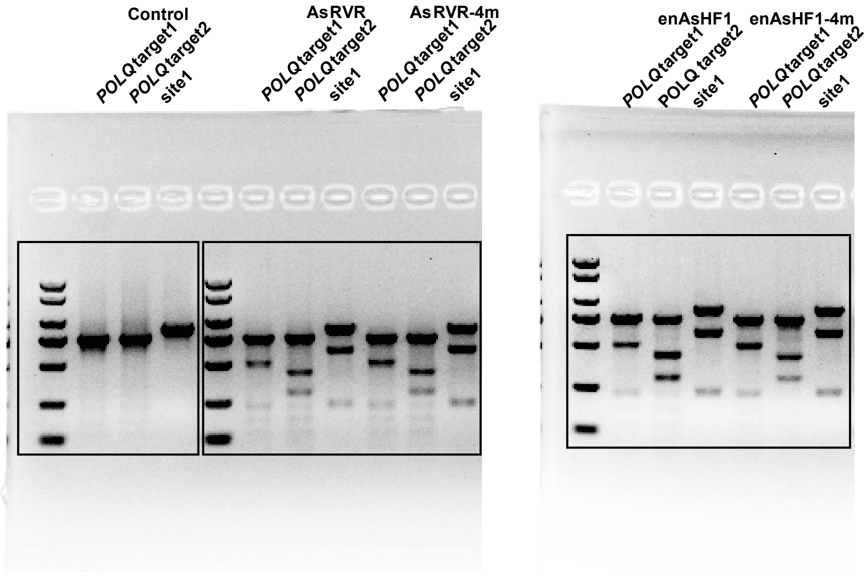

Extended Data Fig. 13: Uncropped gels for Fig 4C and S12F Fig.

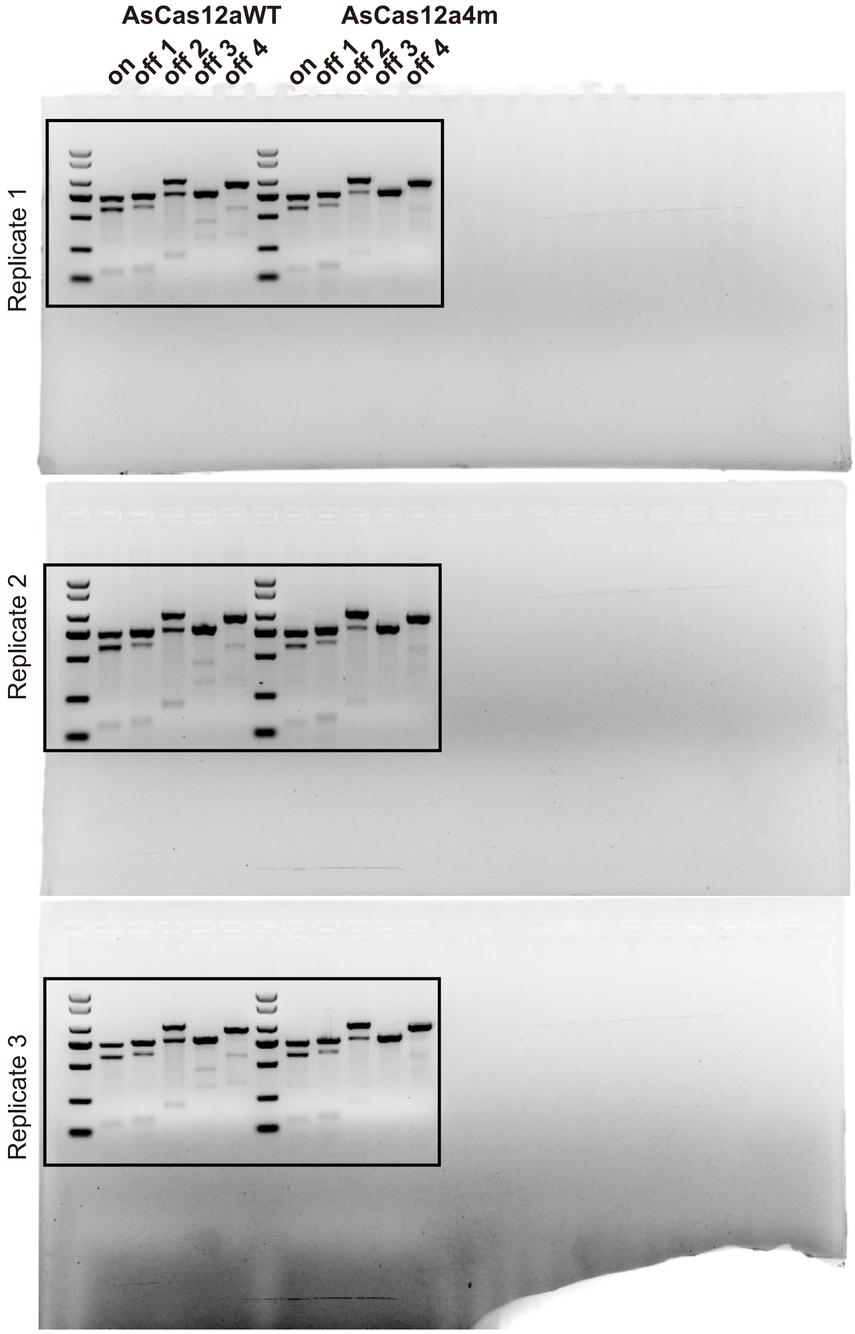

Extended Data Fig. 14: Uncropped gels for Fig 5A.

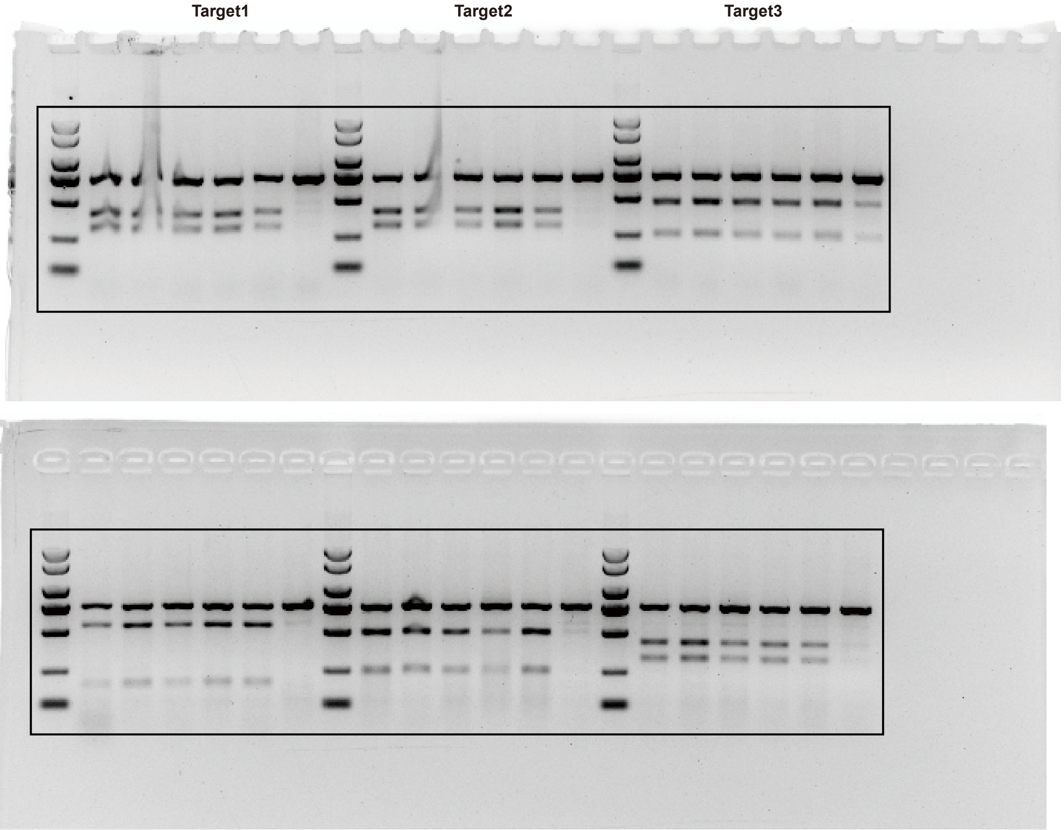

S186	−	A	A	A	A	A	−	A	A	A	A	A	−	A	A	A	A	A
R301	−	A	A	A	A	A	−	A	A	A	A	A	−	A	A	A	A	A
T315	−	A	A	A	A	A	−	A	A	A	A	A	−	A	A	A	A	A
Q1014	−	A	A	A	A	A	−	A	A	A	A	A	−	A	A	A	A	A
K414	−	−	A	−	−	−	−	−	A	−	−	−	−	−	A	−	−	−
S376	−	−	−	A	−	−	−	−	−	A	−	−	−	−	−	A	−	−
Q286	−	−	−	−	A	−	−	−	−	−	A	−	−	−	−	−	A	−
W382	−	−	−	−	−	A	−	−	−	−	−	A	−	−	−	−	−	A

Extended Data Fig. 15: Uncropped blot for S13C Fig.

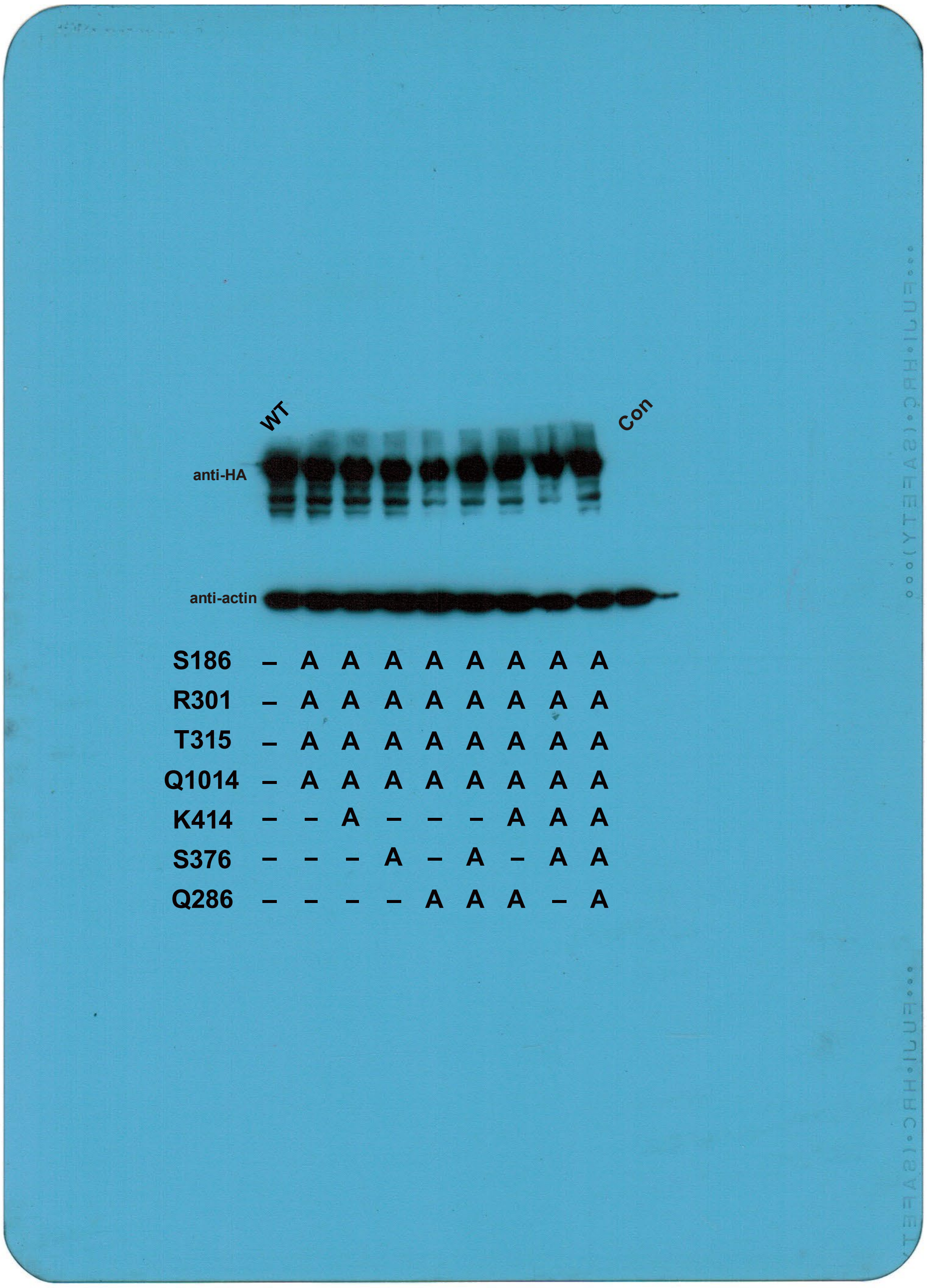

Extended Data Fig. 16: Uncropped gels for S13D Fig.

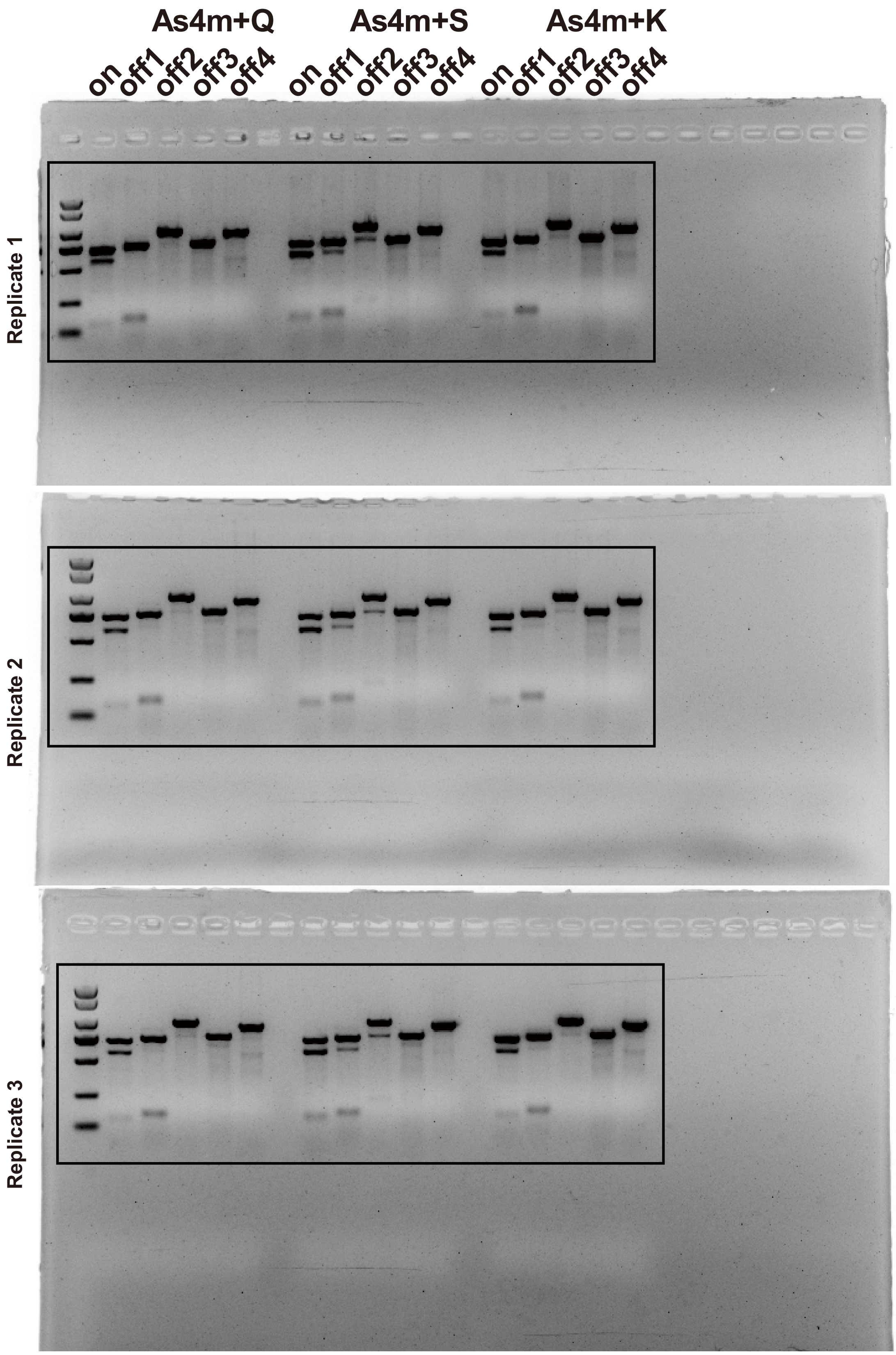

Extended Data Fig. 17: Uncropped gels for S13E Fig.

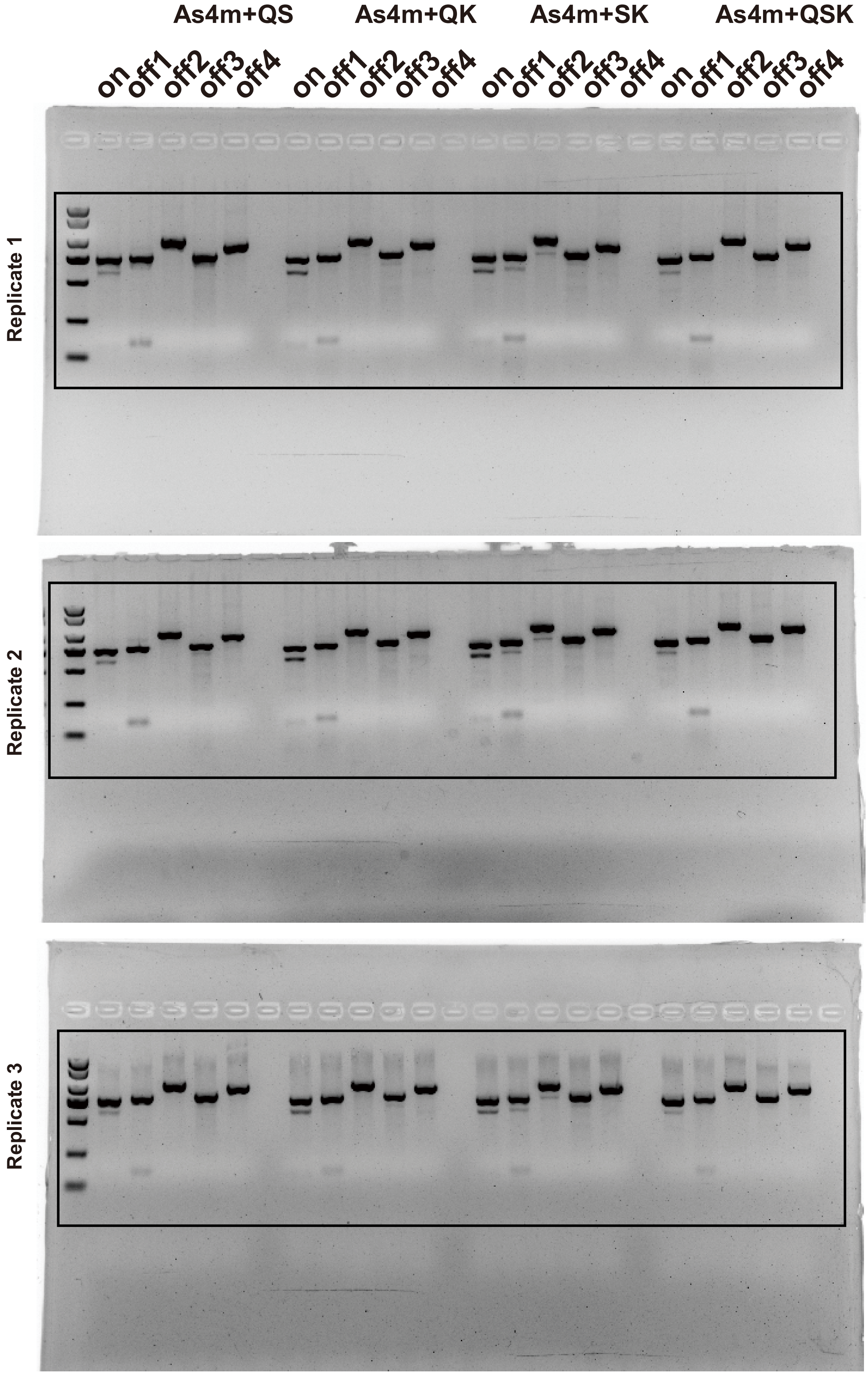

Extended Data Fig. 18: Uncropped gels for S14 Fig.

A

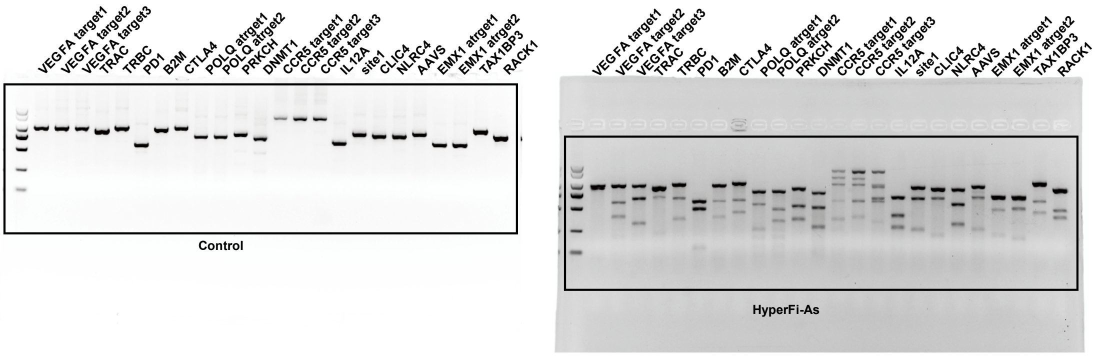

C

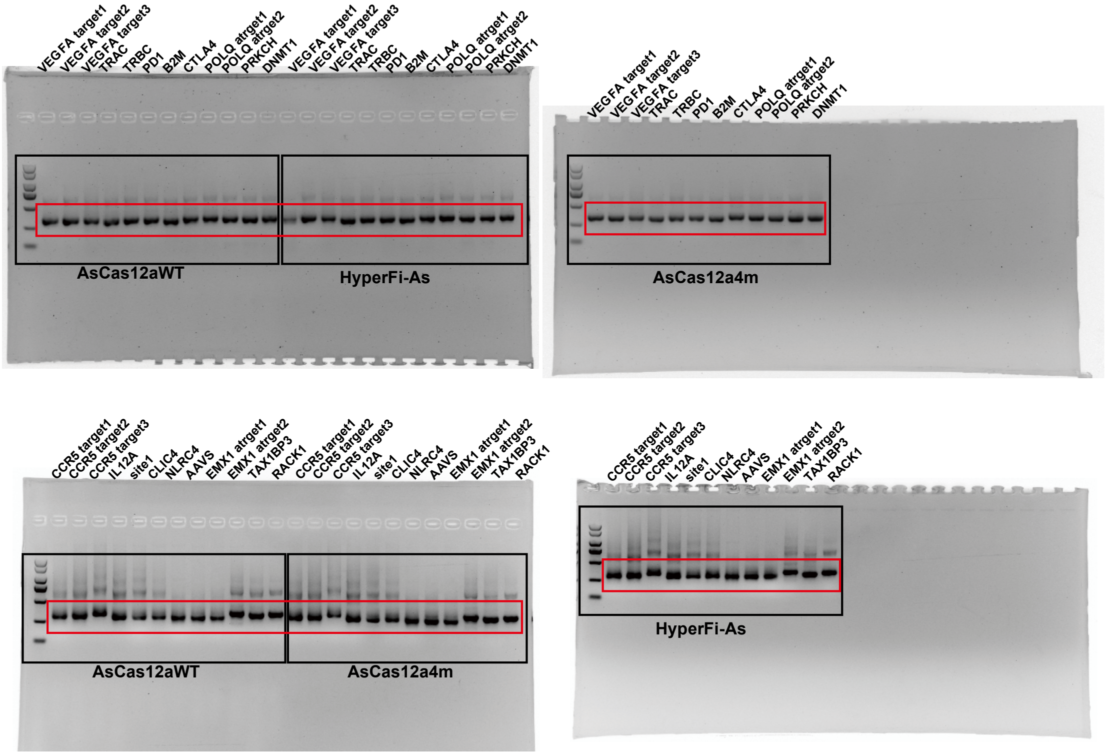

Supplement: S1 Raw Images — (PDF) [file pbio.3002514.s024.pdf]
