# Supplementary material for: Engineering of Cas12a nuclease variants with enhanced genome-editing specificity
Source: PLoS Biol. 2024 Mar 14;22(3):e3002514. doi: 10.1371/journal.pbio.3002514 (PMC10965058; doi:10.1371/journal.pbio.3002514)
Supplement: Attachment — Submitted filename: Response to Reviews.docx [file pbio.3002514.s025.docx]

Dear Editor and Reviewers:

Thank you very much for the letter and the reviewers’ comments concerning our manuscript entitled “Rational designs of Cas12a nuclease with the improved targeting specificity at both the proximal and distal region from the PAM” (ID: PBIOLOGY-D-23-01543R1). Those comments are all very valuable and very helpful for revising and improving our paper. We have read the comments very carefully and all the comments were addressed. Revised portions are using track changes in the paper. Below we will provide point-by-point responses to the reviewers’ comments.

Reviewer comments:

Reviewer #1: Chen et al. developed engineered versions of AsCas12a by introducing alanine substitutions to residues involved in the interaction with sugar and phosphate groups of the target DNA and crRNA. The resulting AsCas12a variants, As4m and HyperFi-As, demonstrated enhanced genome editing specificity compared to the wildtype AsCas12a. However, the genome editing efficiencies of As4m and HyperFi-As at on-target sites have not been definitively evaluated. Furthermore, additional modifications are required to clarify the manuscript for a broader readership. Therefore, I strongly recommend that the authors address these points to further improve the manuscript.

*1. A comprehensive evaluation of the general activity of As4m and HyperFi-As, as well as the wildtype AsCas12a, is needed. Considering the inherent tradeoff between on-target activity and specificity in most Cas9 variants, it is important to examine this in detail. Evaluating the indel frequency at a minimum of 20-30 endogenous sites would be highly suitable for this purpose. Additionally, I strongly recommend utilizing deep sequencing instead of the T7E1 assay, to enhance the reliability of the findings.*

Response:

The reviewer has made a good suggestion and we totally agree with the reviewer that using deep sequencing to evaluate the indel frequency. We detected another 24 endogenous target sites including *TRAC*, *TRBC*, *AAVS*, *B2M*, *PD1*, *CTLA4*, *PRKCH*, *DNMT1*, *NLRC4*, *CLIC4*, *IL12A*, *EMX1*, *TAX1BP3*, *RACK1* (**Response Fig. 1A)**. We used T7E1 assay to conform the genome editing activity of HyperFi-As (**Response Fig. 1A)**. Moreover, indel frequency assessed by deep sequencing (**Response Fig. 1B, C)**. AsCas12a4m, HyperFi-As showed 95% and 90% of the on-target cleavage efficiencies compared to AsCas12aWT at the same sites (**Response Fig. 1D)**.

**Response** **Figure 1:** Evaluation of the activity of AsCas12aWT, AsCas12a4m and HyperFi-As. **A**, Validation of HyperFi-As performance at 24 endogenous target sites with TTTV (V = A, C, or G) PAMs in HEK293T cells. **B**, Schematic of the deep sequencing library constructs. Red arrows indicate genome specific primers with Illumina P5 and P7 adapter sequences. PCR library is about 280 bp. **C**, Full gel images of AsCas12aWT, AsCas12a4m and HyperFi-As deep sequencing libraries. Red box indicated the library band of each endogenous target sites. **D**, Comparison of the activity of AsCas12aWT, AsCas12a4m and HyperFi-As in HEK293T cells. Each dot represents a target site. Indel frequency assessed by deep sequencing. The median and interquartile range are shown.

*2. In Figure 3D and related figures, quantitatively evaluating genome editing efficiency using the read count from Guide-seq poses challenges. To validate the off-target sites identified by Guide-seq, it is necessary to confirm the occurrence of indels at the endogenous sites using deep sequencing.*

Response:

Thanks a lot for good advice. I realized that this review was supposed to refer to Figure 2D not Figure 3D. To validate the off-target sites identified by GUIDE-seq in Figure 2D (**Response** **Figure 2A)** and Figure 3E (**Response** **Figure 2B)**, we confirmed the occurrence of indels at the endogenous sites using deep sequencing. AsCas12a4m generated low levels of nonspecific editing at the off-target sites identified by GUIDE-seq, and an improvement of approximately 1.1- to 11-fold compared to AsCas12aWT (**Response** **Figure 2A)**. We also compared the performance of enAsHF1, AsRVR and enAsHF1-4m, AsRVR-4m at 3 endogenous on-target sites and 23 endogenous off-target sites identified by GUIDE-seq. As a result, enAsHF1, AsRVR, enAsHF1-4m and AsRVR-4m showed enough cleavage activities at 3 endogenous on-target sites (*POLQ*1/2, site1) (**Response** **Figure 2B)**. Moreover, enAsHF1-4m and AsRVR-4m caused significant decreases in off-target sites cleavages, and an improvement of approximately 1- to 55-fold and 1- to 73-fold compared to enAsHF1 and AsRVR (**Response** **Figure 2B)**.

**Response** **Figure 2:** Deep sequencing validation the off-target sites identified by GUIDE-seq. **A**, Percent modification of on-target and GUIDE-seq detected off-target sites with indel mutations for AsCas12aWT and AsCas12a4m. **B**, Percent modification of on-target and GUIDE-seq detected off-target sites with indel mutations for enAsHF1, AsRVR, enAsHF1-4m and AsRVR-4m. On-target cleavages of these variants at 3 endogenous sites evaluated by T7 endonuclease I assay (gel images). Mismatched positions within the spacer or PAM are highlighted in red. Indel frequency assessed by deep sequencing. Error bars represent s.e.m. for n=2.

*3. Figure 5 lacks completeness and contains multiple inaccuracies, making it difficult for readers to understand. The description in the main text does not match the results shown in Fig 5B and Fig 5G. Additionally, Fig 5D is not mentioned anywhere in the manuscript.*

Response:

We are very grateful to your kind reminder. We have modified the main text about Figure 5 carefully. Multiple inaccuracies have been clarified and “Changes Highlighted” can be found in a revised version.

*4. Additional experiments are required to compare the specificity of SpCas9, AsCas12a, and their derived variants since on-target activity can affect specificity. Although the target site chosen by the authors contained PAM sequences for both SpCas9 and AsCas12a, the spacer sequences are in opposite orientations, resulting in position-dependent differences in nucleotide composition, which may lead to variations in on-target activity. Therefore, it is necessary to investigate whether SpCas9, AsCas12a, and their variants* *exhibit similar indel frequency values at the on-target sites.*

Response:

Thank you very much for valuable advices. To evaluate the cleavage activity of AsCas12a, SpCas9, and their derived variants, we performed T7E1 assay at 7 endogenous target sites contained PAM sequences for both SpCas9 and AsCas12a (**Response** **Figure 3A)**. As shown in **Response** **Figure 3B, C**, SpCas9, AsCas12a, and their variants, except for SuperFi-SpCas9 exhibited similar indel frequency values at the on-target sites, which was consistent with the description in our manuscript main text.

**Response** **Figure 3:** Comparison gene-editing efficiencies of AsCas12a, SpCas9 and their derived variants. **A**, On-target sites contained PAM sequences for both SpCas9 and AsCas12a. **B**, Activity of AsCas12a, SpCas9 and their derived variants on targets with candidate PAM sequences measured by T7 endonuclease I assay. **C**, Average activity of AsCas12a, SpCas9 and their derived variants on targets; Summary of on-target modifications from **B**.

*5. Further explanations and revisions are needed for the interpretation of the results in Figure 6. It would be helpful to provide additional explanations about the meaning of state 1, 2, and 3, as well as the transitions from state 1 to 2 and from state 2 to 3. The y-axis in Figure 6D is unclear and hinders understanding of the content. Is it the ratio of RNP molecules that do not form R loop complexes? Furthermore, there is a need to clarify the meaning of terms such as "instability." Lastly, the addition of a figure showing the state transitions of WT AsCas12 and the variants, similar to Fig 6B, would be beneficial.*

Response:

Thanks a lot for good advices.

(1). The difference between States 1, 2, and 3 is the length of the non-target sequence bound to Cas12a. In State 1, the Cas12a-crRNA-DNA complex forms a tight binding, encompassing both the target sequence and the non-target sequence. State 2 represents a partial disassociation of the Cas12a-crRNA-DNA complex, with the non-target strand upstream of the PAM being released from the R-loop complex while the strand on the left side of the hairpin remains bound. In State 3, the hairpin downstream of the PAM, including the PAM sequence, unfolds under force and is released from the Cas12a-crRNA complex. The transition from State 1 to State 2 means the non-target strand upstream of the PAM moving away from the R-loop complex, indicating a relatively weaker binding. The transition from State 2 to State 3 represents the unfolding of the hairpin strand downstream of the PAM including PAM sequence.

(2). Figure 6 reveals that HyperFi-As exhibited similar gene editing efficiency at on-target sites but displayed lower tolerance for off-target sites compared to AsCas12aWT. When the DNA extension reached state 3, the hairpin completely unzipped, and the non-target strand is released from crRNA-Cas12 complex. We then calculated the normalized percentage of unzipping, denoted as “p = M/N”, where “M” represents the number of cycles in which the hairpin unzipped completely, and “N” is the total number of cycles. This ratio signifies the proportion of RNP molecules that do not form R-loop complexes

(3). In this context, “instability” refers to the possibility of the non-target sequence to unzip completely and move away from the crRNA-Cas12 complex. Simultaneously, the faster the hairpin extension reaches state 3, the less stable the complex is.

(4). We have included a figure that illustrates the state transitions of AsCas12WT and its variants. The key distinction between these two types of Cas12a enzymes lies in the time required to reach State 3 (**Response** **Figure 4**).

**Response** **Figure 4:** The state transitions of AsCas12WT and HyperFi-As.

*6. The references need overall organization and revision. (Line 40-41) Reference 27 is not about eSpCas9, and Reference 29 is about SaCas9, so the corresponding sentences need to be modified. (Line 56) Reference 15 cannot provide support for the specificity of AsCas12a. Throughout the manuscript, preceding studies on the* *structural analysis of AsCas12a are also not cited.*

Response:

We are very sorry about our carelessness. We have revised the incorrect references in the right order. The missing reference about the structural analysis of AsCas12a were cited in the right place.

*Minor concerns:*

*(Line 110) "wild-type SpCas9" should be changed to "wild-type AsCas12a."*

*(Line 160) There is inconsistency in the labeling of the AsRVR and enAsHF1 experimental groups between the manuscript and Figure 3, making it difficult to understand the content.*

*(Line 214) The manuscript and Figure 5 have different labels for W382 and W342, so correction is needed.*

Response:

We are very grateful to your kind reminder.

(Line 110) We have changed "wild-type SpCas9" to "wild-type AsCas12a.".

(Line 160) We have relabeled the AsRVR and enAsHF1 experimental groups between the manuscript and Figure 3.

(Line 214) We have changed W342 to W382 in Figure 5.

Reviewer #2: CRISPR-Cas12a/Cpf1 enzymes are naturally high specificity nucleases that offer advantages over Cas9-based systems. Although one can argue whether their off-target potential is a problem worth solving (given the generally low levels of off-targets), engineered enzymes that preserve on-target editing while minimizing off-target edits are advantageous for obvious reasons. Here, Chen et al. seek to develop higher-fidelity Cas12a enzymes that have reduced off-target editing compared to the several other versions previously described. This manuscript takes a nearly identical blueprint to that described for SpCas9-HF1 (PMID: 26735016) with similar figures and assays. The result is a highly efficient and specific Cas12a enzyme that has reduced off-target potential. The manuscript is well written, the HyperFi-As enzyme appears to be quite useful. and the data presentations are generally clear; thus, I have few comments.

Comments:

*1. Naturally high fidelity. Some additional commentary from the authors about whether this is a problem worth solving would be helpful. The author's own data shows that in most cases there are either no off-targets, or very few at 10- or 100-fold lower levels than the on-target site. These results are consistent with many prior reports that Cas12a is naturally a high specificity enzyme. This referee is not suggesting that this isn't an important problem, but the authors should more clearly describe why even low level off-targets may be a concern.*

Response:

We totally agree that Cas12a is naturally a high specificity enzyme. As far as we known, there is no clear clue that what kinds of low off-targeting is enough for therapies. That is why we want to further chase low off-targeting and choose Cas12a as the candidate instead of Cas9. We think the off-targeting in the genome level leads to big risks. And the lower off-targeting we can get, the more safety for therapies we can have.

*2. On-target activity. The authors's data show that in general HyperFi-As tends to retain on-target editing. Under what conditions were these assays performed (presumably high levels of plasmid delivery into HEK 293 cells)? Some discussion about the likelihood of the enzyme maintaining on-target efficiency in different contexts (e.g. mRNA or RNP delivery) would be helpful. If the authors have data to show that HyperFi-As works well in the mRNA or RNP format, it would be nice to add this data to the manuscript.*

Response:

We are very grateful to your kind advices. We performed all on-target editing assay at the same condition. In brief, approximately 1.2 × 10^5^ cells were seeded into per well of 24-well, plate a day before transfection, 600 ng Cas12a and 300 ng crRNA expression plasmids were transfected into cells using Hieff Trans Liposomal Transfection Reagent. 48 hours post-transfection, cells were collected and lysed in 50 μl Lysis buffer. For T7E1 assay, using 1 μl cell lysis as template to amply the target genomic region. PCR products were purified and the concentrations were determined. 250 ng of purified PCR products were used to perform T7E1 cleavage assay. For deep sequencing, using 1 μl cell lysis as template to amply the target genomic region (**Response Fig. 1B**). PCR products were purified and the concentrations were determined. The sequencing library sequenced on an Illumina Instrument and data was analyzed using crispresso2.

We agree with the Reviewer that using mRNA and RNP would be stronger to support the maintaining on-target efficiency claim. But we do not specialize in mRNA and RNP. We may not be able to complete these experiments within the allotted time. We will do these in our future studies.

*3. Is there a way that GUIDE-seq is missing off-targets for Cas12a or other enzymes that leave staggered breaks?*

Response:

Thank you very much for good questions. GUIDE-seq was reported to detect off-target sites that are mutagenized by RGNs (CRISPR RNA-guided nucleases) with frequencies as low as ~0.1% sensitively [1]. Small number of dsODN (double-stranded oligodeoxynucleotide) integration at these breaks by means of an end-joining process consistent with NHEJ may lead to some off-target sites missing.

*4. Some of the other improved specificity Cas12a enzymes that have been developed should be discussed earlier in the manuscript (briefly in the introduction). Many of these papers are mentioned later in the results, but since this work is continuing a long line of efforts by other groups, it would be helpful for the authors to add more context. For instance, AsCas12a Plus from Zhili Rong's group (35468792), the HF variant from Joung's group (PMID 30742127), unpublished but public information on AsCpf1 from Joung's group (https://patents.justia.com/patent/20220025347), AsCas12a Ultra (34162850) and others. This prior work should be mentioned in the introduction, given that there is some overlap for the amino acid substitutions selected in this current work.*

Response:

Thanks a lot for good advices. We have added discussion about these improved specificity Cas12a enzymes in the introduction. The related references have been cited in the right places.

*5. The R-loop stability data is nice. Does this mean that the HyperFi-As enzyme may not work as well as a base editors? Or that it will be extra specific as a base editor?*

Response:

We are very grateful to your comments for our R-loop stability data. It is not clear whether HyperFi-As can function as a base editor like AsCas12aWT or will be extra specific as a base editor. In this stage our R-loop stability data can just indicates that HyperFi-As has the same stability as AsCas12aWT on R-loop in the absence of mismatch. In the absence of mismatch, HyperFi-As is indicated to be more easily dropped and unstable than WT AsCas12a.

*6. Citations are missing for many statements in the introduction and throughout the manuscript. For example:*

*a. Line 47 - PMID: 26422227*

*b. Line 49 - PMIDs: 26422227, 27918548, 27096362, 29083402, etc.*

*c. Line 51 - PMIDs: 27272384, 27347757, etc.*

*d. Etc*

Response:

We are very sorry for our omission of these citations. We have added related citations in the right places.

Reviewer #3: In this manuscript, Chen et al. report their work on the structure-guided engineering of AsCas12a to enhance the specificity for genome editing in human cells. Through alanine substitution of residues involved in interaction with the target DNA and crRNA at the PAM-distal end, they have made an AsCas12a variant with five point mutations S186A/R301A/T315A/Q1014A/K414A (named HyperFi-As). Their experiments demonstrate that this variant maintains on-target activity while reducing off-target effects.

*Overall, the topic of reducing off-target effects is of significant interest in the genome editing field. This study contributes valuable data by analyzing Cas12a and comparing it with various other Cas12a and Cas9 variants aimed at addressing the same challenge. The strength of this research lies in this comparative analysis. However, my primary concern is the extent of improvement offered by this new Cas12a variant relative to previously reported ones. Although the authors claim their Cas12a variant to be superior, the robustness of this variant is unclear. It remains to be seen whether the reduced off-target rate is applicable across other cell lines. While I am not necessarily asking the authors to test additional cell lines in this paper, although such an effort would be creditable, it would be beneficial to situate their findings within a broader context. Discussion of the limitations of their work, like a more quantitative analysis of the observed improvement and the need for testing more cell lines, would be welcome.*

Response:

We are very grateful to your kind advices. In the manuscript, we comprehensively assessed these variants' capability to reduce genome-wide off-target effects in human 293T cells by using GUIDE-seq. We counted the total number of genome-wide off-target cleavage sites identified by GUIDE-seq for these variants with gRNAs targeted to 5 endogenous sites (**Response** **Figure 5A, B**). In summary, GUIDE-seq analysis of HyperFi-As showed that the percent off-target effects decreased by 81.8%, 89.7%, 78%, 42.2%, 98.6%, compared to As, As ultra, As plus, SuperFi-SpCas9, SpCas9 (**Response** **Figure 5C**).

We agree with the Reviewer that testing additional cell lines would be beneficial to situate our findings. Cas12a variants such as As plus, Lb2K548R have been proven to possess high fidelity performance in different cell lines [5,6]. In this paper, we spent a lot of time verifying the high-fidelity performance of the HyperFi-As in many endogenous sites and compared it with various high specify Cas12a and Cas9 variants for comparative analysis, and little time was left for us to test additional cell lines. We will definitely test this good point in our future studies.

**Response** **Figure 5:** Assessment off-target effects of CRISPR-Cas nucleases. **A**, On-target sites contained PAM sequences for both SpCas9 and AsCas12a. **B**, Histograms illustrating the total number of GUIDE-seq detected off-target sites for As, As ultra, As plus, SuperFi-SpCas9, SpCas9. **C**, Histograms illustrating the percent off-target effects of HyperFi-As compared to other variants.

*In addition, I have the following specific comments for the authors' consideration.*

*1) Page 3, line 41: The authors provide several examples of structure-guided protein engineering of Cas9. Could they briefly summarize the principles utilized for the engineering? Were the residues involved in binding the PAM-distal end of target DNA and crRNA also mutated?*

Response:

Principles utilized for engineering for these examples were described as follows briefly.

SpCas9-HF1 (The interaction between the protein and the target DNA is weakened, and the retaliatory force between the DNA double strands is enhanced, which requires better pairing between the target DNA and crRNA), HypaCas9 (Reduce the energy threshold of HNH domain flipping, DNA and RNA can be flipped for cutting when they are normally paired, and cannot be flipped for cutting if there is mismatch), eSpCas9 (principle same as SpCas9-HF1, only change target DNA to Non-target DNA), HeFSpCas9 (It incorporates all mutations of SpCas9-HF1 and eSpCas9), SaCas9-HF1 (principle same as SpCas9-HF1, only change SpCas9 to SaCas9).

We were the first to modify the interaction of the PAM-distal end of crRNA strand with amino acids to obtain a high-fidelity AsCas12a variant (HyperFi-As).

*2) Page 4, line 50: The authors claim that Cas12a exhibits low off-target effects. Is this in comparison to wild type Cas9? They attribute the low off-target activity to 'low* *crRNA-DNA mismatch tolerance', but do not provide citations. Could they elaborate on the reasoning behind the low off-target effects in Cas12a?*

Response:

Yes, this is in comparison to wild type Cas9. We are sorry to miss the citation of this statement. We have added related citation in the right place. The reason behind the low off-target effects in Cas12a might be complicated. One of the major reasons could be the relative weak binding stability of Cas12a to the crRNA-DNA loop. This relative weak binding stability might be in a proper range which barely decrease the gene editing efficacy at on-target sites. At off-target sites the mismatch of crRNA-DNA might greatly weaken the binding stability and be less tolerant. Subsequently less editing would happen here.

*3) Page 8, line 80: When referencing 'our previous work', please provide a citation. The same applies to 'our former experiment' on page 10.*

Response:

We have added related citation in the right place.

*4) Page 6, lines 91-101: Could the authors provide a more quantitative description of the results? When they say 'dramatically', it is unclear to the reader the extent of the difference observed. Consider using more quantitative description for a few other results as well, for example, 'showed enough cleavage events' on page 11.*

Response:

Thanks a lot for your good advices. We have provided a more quantitative description of the results in the main text. And “Changes Highlighted” can be found in a revised version.

*5) Page 7, line 121: Could the authors explain how they measured cleavage efficiency in human cells? It seems to me that direct cleavage is not directly observable. Instead, the indel rate might be an indirect reflection of cleavage. If my understanding is correct, please revise the text for better accuracy.*

Response:

T7 endonuclease I (T7E1) catalyzes the cleavage of DNA mismatches, and has been widely used to detect gene editing activity [2-4]. We performed T7E1 assay to measured cleavage efficiency in human cells. In brief, approximately 1.2 × 10^5^ cells were seeded into per well of 24-well, plate a day before transfection, 600 ng Cas12a and 300 ng crRNA expression plasmids were transfected into cells using Hieff Trans Liposomal Transfection Reagent. 48 hours post-transfection, cells were collected and lysed in 50 μl Lysis buffer.

For T7E1 assay, using 1 μl cell lysis as template to amply the target genomic region. PCR products were purified and the concentrations were determined. 250 ng of purified PCR products were mixed with 1 μl 10×T7E1 buffer (Vazyme) and ultrapure water to a final volume of 10 μl, and subjected to a re-annealing process to enable heteroduplex formation: 95 °C 397 for 3 min, 95 °C for 30 s, 90 °C for 30s, 85 °C for 30 s, 80 °C for 30 s, 75 °C for 30 s , 70 °C for 30 s, 65 °C for 30 s, 60 °C for 30 s, 55 °C for 30 s, 50 °C for 30 s, 45 °C for 30 s, 40 °C for 30 s, 35 °C for 30 s, 30 °C for 30 s, and 25 °C for 1 min. After re-annealing, products were treated with T7 Endonuclease I (EN303-01/02, Vazyme) for 15 min at 37 °C. The reaction mixtures were run on 2% agarose gels and imaged with ChemiDocTM XRS+and analyzed according to strip intensities. Indel percentage was determined by the formula: 100 × (1 – sqrt (b + c)/(a + b + c)), where a is the integrated intensity of the undigested PCR product and b and c are the integrated intensities of the cleavage product.

*6) Page 12, line 221: I recommend using 'indels' instead of 'in/dels', although the decision is up to the authors.*

Response:

Thanks a lot for your good advice. We adopted this recommendation, and changed 'in/dels' to 'indels'. “Changes Highlighted” can be found in a revised version.

*7) Page 13, line 260: There is a typo in 'intermediate states' (not 'intermediated states').*

Response:

Thanks a lot for your kind reminder. We have modified this typo. “Changes Highlighted” can be found in a revised version.

*8) Page 14, line 276: As far as I understand, 'the unzipping of PAM' is not a step in Cas12a activation and is therefore confusing here.*

Response:

Thanks a lot for your good comments. We agree that 'the unzipping of PAM' is not a step in Cas12a activation. The state of the unzipping of PAM was used to suggest how stable the RNP complex of Cas12a could be. The single-molecule experiment here utilized dCas12a to investigate the stability of R-Loops in the presence and absence of mismatches. We observed that the dissociation of the non-target strand from the complex occurs in two steps. In the first step, the non-target strand upstream of the PAM region detaches from the complex under the influence of applied force. This detachment occurs while the PAM region and its subsequent hairpin section remain folded due to their interaction with the protein. However, with prolonged application of force, the region including the PAM and the downstream chain eventually unzipping, this process is unrelated to Cas12a activation.

*9) Page 15, line 295: When indicating that Cas12a unwanted mutation rates are lower than those of Cas9, please provide references.*

Response:

Thanks a lot for your kind reminder. We have added related citation in the right place.

*10) I recommend using 'AcCas12aWT' instead of 'AsWT' and 'AsCas12a4m' instead of 'As4m'.*

Response:

Thanks a lot for your good advice. We adopted this recommendation, and changed 'AsWT' to AsCas12aWT', and 'As4m' to AsCas12a4m ' in main text. “Changes Highlighted” can be found in a revised version.

Special thanks to you for your good comments.

**References**

1. Tsai SQ, Zheng Z, Nguyen NT, Liebers M, Topkar VV, Thapar V, et al. GUIDE-seq enables genome wide profiling of off-target cleavage by CRISPR-Cas nucleases. Nat Biotechnol. 2015;33(2):187-97. Epub 2014/12/17. doi: 10.1038/nbt.3117. PubMed PMID: 25513782; PubMed Central PMCID: PMCPMC4320685.

2. Kleinstiver BP, Pattanayak V, Prew MS, Tsai SQ, Nguyen NT, Zheng Z, et al. High-fidelity CRISPR-Cas9 nucleases with no detectable genome-wide off-target effects. Nature. 2016;529(7587):490-5. Epub 2016/01/07. doi: 10.1038/nature16526. PubMed PMID: 26735016; PubMed Central PMCID: PMCPMC4851738.

3. Kim D, Kim J, Hur JK, Been KW, Yoon SH, Kim JS. Genome-wide analysis reveals specificities of Cpf1 endonucleases in human cells. Nat Biotechnol. 2016;34(8):863-8. Epub 2016/06/09. doi: 10.1038/nbt.3609. PubMed PMID: 27272384.

4. Kleinstiver BP, Tsai SQ, Prew MS, Nguyen NT, Welch MM, Lopez JM, et al. Genome-wide

specificities of CRISPR-Cas Cpf1 nucleases in human cells. Nat Biotechnol. 2016;34(8):869-74. Epub 2016/06/28. doi: 10.1038/nbt.3620. PubMed PMID: 27347757; PubMed Central PMCID: PMCPMC4980201.

5. Huang H, Huang G, Tan Z, Hu Y, Shan L, Zhou J, et al. Engineered Cas12a-Plus nuclease enables gene editing with enhanced activity and specificity. BMC Biol. 2022;20(1):91. Epub 2022/04/27. doi:10.1186/s12915-022-01296-1. PubMed PMID: 35468792; PubMed Central PMCID: PMCPMC9040236.

6. Zhou J, Chen P, Wang H, Liu H, Li Y, Zhang Y, et al. Cas12a variants designed for lower genome wide off-target effect through stringent PAM recognition. Mol Ther. 2022;30(1):244-55. Epub 2021/10/24. doi: 10.1016/j.ymthe.2021.10.010. PubMed PMID: 34687846; PubMed Central PMCID: PMCPMC8753454.
