# Supplementary material for: Engineering of Cas12a nuclease variants with enhanced genome-editing specificity
Source: PLoS Biol. 2024 Mar 14;22(3):e3002514. doi: 10.1371/journal.pbio.3002514 (PMC10965058; doi:10.1371/journal.pbio.3002514)
Supplement: Attachment — Submitted filename: Response to Reviews-R2.docx [file pbio.3002514.s026.docx]

Dear Editor:

We greatly appreciate your letter and the reviewers' comments on our manuscript entitled "The Cas12a ortholog with stringent PAM recognition followed by low off-targeting in genome editing" (ID: PBIOLOGY-D-23-01543R2). Thank you very much for the kind title change. In the resubmission, we have added all the additional data and discussions to the manuscript as requested by the editor and reviewers. Below we provide point-by-point responses to reviewers' comments.

*Reviewer #2: I feel that the Authors have made inadequate revisions to their manuscript. The authors should not just respond to the Referee comments in their rebuttal letter - they must adjust their manuscript to clarify their text in ways that reflect the requests of the Referees. The Referees have asked various questions for very specific reasons to help improve the clarity of the study - not just for a response in the rebuttal. These points need to be made more clear throughout the manuscript. Please adjust the text accordingly in light of all initial reviewer comments.*

Response:

We are very grateful for your kind reminder. We have changed the main text and adjusted the text accordingly in light of all the initial reviewer comments. Please see "Changes Highlighted" for a revised version.

Some separate comments:

*1. Lines 58/59 - This modification is incorrect. AsCas12a high-fidelity variants have been developed to reduce off-target editing* *at unwanted loci, not "to decrease the indels even at the spacer region", which implies the on-target site.*

Response:

We are very grateful for your kind reminder. We have corrected this incorrect description on lines 58-59 in the revised version.

*2. One experiment the authors could do to simulate lower expression levels / more transient enzyme exposure (as would be observed by RNP or mRNA delivery) would be to titrate their plasmid delivery down to see whether the engineered enzyme has poorer on-target activity compared to wild-type. In your Discussion, please add some discussion of the translatability of these enzymes into different delivery formats.*

Response:

Thank you very much for the valuable advice. We transfected HEK293T cells with different amounts of AsCas12a WT and HyperFi-AsCas12a plasmids. After 48 hours of incubation the genomes were extracted and subjected to the T7E1 assay. The results indicate that both exhibit comparable editing activities (**Response Figure 1**). This figure has been added in the revision of **Fig S14**. A discussion on the different delivery formats of these enzymes has been added to the revised manuscript. "Changes Highlighted" can be found on lines 403-410 in the revised version.

**Response** **Figure 1:** Evaluation of the activity of AsCas12aWT and HyperFi-AsCas12a with different amounts of plasmid.

*3.* *In your Discussion, add some text to discuss why you think GUIDE-seq is an adequate assay to detect Cas12a off-target sites (and could cite prior work). This is frequently discussed in the genome editing field and should be a point of discussion in this work given how many conclusions are dependent on the results of the GUIDE-seq assay.*

Response:

Thanks a lot for the good advice. We have added some text to discuss GUIDE-seq. "Changes Highlighted" can be found on lines 338-343 in a revised version.

4. *In the results section when the authors describe which amino acid substitutions they are using in their modified enzyme(s), they should list specific fidelity-enhancing mutations that have been described by prior groups in published literature, so that the readership can best understand what mutations are new compared to what mutations have been described by others.*

Response:

We are very grateful for your kind reminder. We have listed specific fidelity enhancing mutations described by previous groups in the Results section. "Changes Highlighted" can be found on lines 89-90 in a revised version.
